# Plasmonic nanotechnology for photothermal applications – an evaluation

**DOI:** 10.3762/bjnano.14.33

**Published:** 2023-03-27

**Authors:** A R Indhu, L Keerthana, Gnanaprakash Dharmalingam

**Affiliations:** 1 Plasmonic Nanomaterials Laboratory, PSG Institute of Advanced Studies, Coimbatore-641004, India

**Keywords:** nanoparticle heating, phonons, photothermal, plasmonic, stability, surface plasmon resonance

## Abstract

The application of plasmonic nanoparticles is motivated by the phenomenon of surface plasmon resonance. Owing to the tunability of optothermal properties and enhanced stability, these nanostructures show a wide range of applications in optical sensors, steam generation, water desalination, thermal energy storage, and biomedical applications such as photothermal (PT) therapy. The PT effect, that is, the conversion of absorbed light to heat by these particles, has led to thriving research regarding the utilization of plasmonic nanoparticles for a myriad of applications. The design of conventional nanomaterials for PT conversion has focussed predominantly on the manipulation of photon absorption through bandgap engineering, doping, incorporation, and modification of suitable matrix materials. Plasmonic nanomaterials offer an alternative and attractive approach in this regard, through the flexibility in the excitation of surface plasmons. Specific advantages are the considerable improved bandwidth of the absorption, a higher efficiency of photon absorption, facile tuning, as well as flexibility in the synthesis of plasmonic nanomaterials. This review of plasmonic PT (PPT) research begins with a theoretical discussion on the plasmonic properties of nanoparticles by means of the quasi-static approximation, Mie theory, Gans theory, generic simulations on common plasmonic material morphologies, and the evaluation processes of PT performance. Further, a variety of nanomaterials and material classes that have potential for PPT conversion are elucidated, such as plasmonic metals, bimetals, and metal–metal oxide nanocomposites. A detailed investigation of the essential, but often ignored, concept of thermal, chemical, and aggregation stability of nanoparticles is another part of this review. The challenges that remain, as well as prospective directions and chemistries, regarding nanomaterials for PT conversion are pondered on in the final section of the article, taking into account the specific requirements from different applications.

## Review

### 1 Introduction

With an ever-increasing demand for energy and the inevitable reduction in the dependency on fossil fuels, global energy demand looks to solar power to be a significant provider for its needs, with various solar power conversion technologies in place and rapidly progressing [1]. Electromagnetic radiation, when interacting with a material can transfer energy to its atoms, eventually converted to heat through a series of energy-loss processes. This conversion of electromagnetic energy into heat is called the photothermal (PT) effect. Early stages of the PT effect were initially observed in semiconductors [2], after which researchers started to explore various material phenomena other than bandgap absorption for heat generation in nanoparticles (NPs), leading to a rapid proliferation of materials for the same. For example, organic materials undergo rapid internal relaxation by the PT effect and are often desired in cancer treatment research as they cause little damage to adjacent healthy tissues due to extremely localized heating [3]. Generally, the reduction of material dimensions to the nanoscale, such as in graphene, carbon nanotubes (CNT) and polymers, leads to an enhancement of the PT effect due to factors such as improved thermal conductivity, tunability of materials for realizing broadband energy absorption, appearance of new mechanisms of photon absorption, and improved prospects of preserving material properties [4–6]. Nanoparticle heating can result also due to the conversion of optical absorption by plasmons into heat. This phenomenon of surface plasmon resonance results from the interaction between electromagnetic radiation and typically high-valence materials, leading to oscillations of the free electrons in it. The decay of these collective oscillations into heat is the plasmonic photothermal (PPT) effect. The absorption characteristics such as the wavelength in plasmon resonance can be tuned and controlled by the properties of the nanoparticle such as size, shape, proximity to other particles, as well as the surrounding medium [6]. Indeed, advancements in such manipulation at the nanoscale has aided the use of plasmonic materials [7–8], such as Au nanoparticles (AuNPs), in photodynamic therapy [9–11]. Metal nanoparticles in general have been extensively explored in PPT applications due to their high free electron density and the possibility of intricate tuning of light absorption [12]. Noble metal nanoparticles with resonances in the UV–vis–IR part of the electromagnetic spectrum are especially researched on for PT applications [13], with excellent reviews on materials for mid-IR applications [14], cancer treatment [15], antibacterial research [16], solar-driven vapour evaporation [16], solar collectors [11,17–18], catalysis [19], clean water production [15], and wearable heaters [20–21], to name a few. This review is on PPT nanoparticle research spanning the conventional options (metals and alloys) as well as materials with induced plasmonic properties, with a special emphasis on their stability in terms of temperature and reactivity. With broad applications in therapy [22–23], laser combined imaging, solar vapour generation [24], and biosensors [25], the global market for PT devices is expected to be a multimillion dollar enterprise by 2025 [26]. This review will focus on concepts such as the theoretical aspects of PPT energy conversion (which influence material selection and design), studies on different classes and morphologies of nanomaterials that have been investigated for different applications of PT conversion, and the thermal and chemical stability of PT nanomaterials, which need to be considered prior to making the final choice. We conclude with a broad perspective on current research, challenges that remain to be solved, as well as prospects in terms of material design and deployment for better exploitation of such nanostructures for PT energy conversion.

### 2 Plasmonics in PT conversion

**Figure 1:**
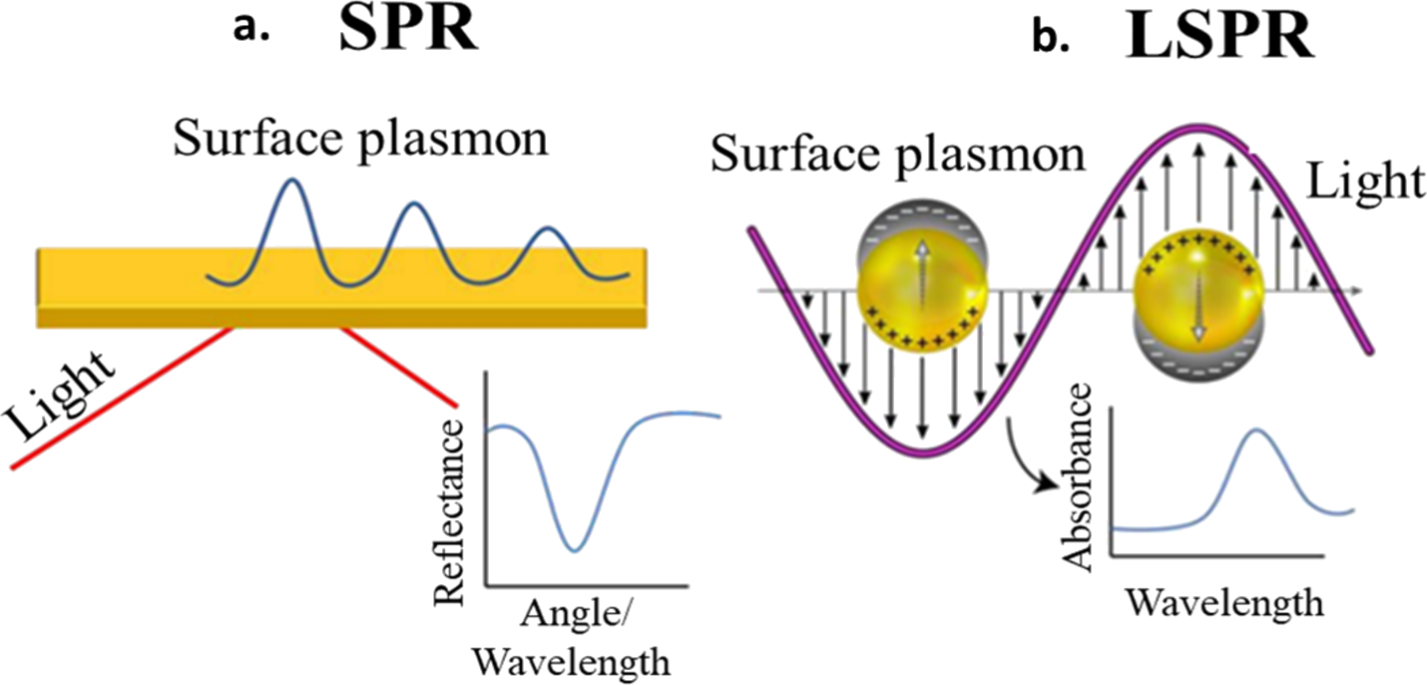
Schematic representation of surface plasmon resonance (SPR) excitation. (a) SPR wave or surface plasmon polariton (SPP). (b) Localized SPR (LSPR) in a spherical nanoparticle and the associated absorbance spectrum. Figure 1a,b was used with permission of The Royal Society of Chemistry, from [31] (“Portable and field-deployed surface plasmon resonance and plasmonic sensors”, J.-F. Masson, Analyst, vol. 145, issue 11, © Copyright 2020); permission conveyed through Copyright Clearance Center, Inc. This content is not subject to CC BY 4.0.

Of the incident radiation from the sun, 8% UV, 42.4% visible light, and 49.6% infrared radiation reach the earth's surface. Applications such as steam generation from solar power can evidently benefit from the use of materials that can absorb as much as possible of the entire spectrum of solar radiation. In this regard, plasmonic nanomaterials with tunable energy absorption can help, and the tunability of such materials only manifests at the nanoscale as changes in the absorption of incident radiation. This tunability is of utmost benefit for PT applications as the region of the electromagnetic spectrum that is not absorbed by generic PT materials can be utilized for absorption and eventual conversion into heat by incorporation of plasmonic nanoparticles of appropriate sizes and shapes. Plasmons, that is, collective electron excitations, are either excited in the bulk of the material (volume plasmons) or on the surface through excitations of the conduction electrons (surface plasmons) as shown in Figure 1. Such excitations, when occurring in nanoparticles, are termed localized surface plasmon resonances (LSPRs) as they are confined within the boundaries of the nanoparticle (in the case of continuous films, they are propagating oscillations termed surface plasmon polaritons (SPPs)). A plasmon in LSPR can be visualized as a quasiparticle confined to the volume of the nanoparticle [27]. The resulting confinement of the absorbed incident electromagnetic radiation within the nanoparticle thus means an effective localization of the incident photon energy, and the decay of this oscillation (through phenomena such as electron–electron, electron–phonon, and electron–surface scattering) releases the absorbed energy into the lattice as heat (or as photons), often making them efficient tunable PT energy materials [24–25]. Interaction of electromagnetic radiation with a material can lead to absorption, transmission, or scattering. Regarding scattering, elastic and inelastic scattering are the major classifications. Elastic scattering means conservation of the photon energy, in inelastic scattering, there are processes other than complete absorption through which photon energy can be transferred to a material. Elastic scattering is not relevant for PT applications as there is no transfer of energy into the material for heating. Absorption/inelastic scattering of electromagnetic radiation can lead to electronic, translation, vibration, and rotational transitions. The interaction time period of electromagnetic radiation with electrons is around 10^−14^ to 10^−15^ s. SPR falls within the regime of electronic transitions and, generally, electronic transitions can be interband as well as intraband transitions. When the energy of the photons is greater than the bandgap, interband transitions are observed. As an example of the energies at which interband transitions [28] occur, Cu, Au, Ag exhibit them at 2.25, 2.4, and 4 eV, respectively, and threshold energy levels of interband transitions are 1.6–1.8 eV for Cu, Au, and Al, as well as 3.5 eV for Ag [29]. Concerning PT applications, radiative transitions such as luminescence and scattering imply inefficiency, as this scattered energy is not converted to heat. Hot electron generation and subsequent thermalization are consequences of SPR absorption that can lead to heat generation, depending on whether the decay of the SPR is through radiative or non-radiative processes. Many metals show plasmonic properties, but for PT applications there is a specific set of requirements including, but not limited to, broadband absorption of electromagnetic radiation, specifically in the UV–vis range (as infrared is already applied for heating), efficient of conversion of the absorbed energy into heat (in contrast to scattering), chemical and physical stability of the nanoparticles (e.g., against agglomeration), ease of synthesis, and low cost. Coinage metals, such as Au, Ag, and Cu, with high densities of free electrons exhibit plasmon resonances in the visible region suitable for PT applications [30]. The subsequent part of this review highlights the parameters that influence various properties of plasmonic materials relevant to PT energy conversion. We first derive the equation quantifying the absorption frequency of plasmons, followed by a discussion on the changes to this frequency that can be induced by changing the governing parameters of this equation, and conclude with a few examples that model the optical scattering properties of generic morphologies such as spheres and nanorods.

#### 2.1 The plasmonic oscillation frequency

The optical response of plasmonic nanoparticles, such as AuNPs, to incident electromagnetic radiation depends on their size, shape, morphology, proximity to one another, as well as the surrounding medium [32]. The vast changes in absorbance due to changes morphology stem predominantly from changes to the directionality of the LSPR (due to changing curvature and dimensionality of the nanoparticle) and changes to the dynamics between the restoring and exciting forces of the plasmons, such as the mean free path, the relative contributions to the plasmon damping of different scattering phenomena, the different scattering processes of the oscillating plasmons, and the screening between the plasmons and the restoring nuclear forces [33–34]. The proximity among LSPR-active nanoparticles is also a major factor. Indeed, combined effects of proximity as well as morphology influence considerably the LSPR properties, for example, in Au nanorods and nanospheres. In contrast, nanospheres and nanorods exhibited considerable tunability of the LSPR due to changes to the localized electromagnetic field of the plasmons due to changing curvature [35]. Finally, changes to the material composition, such as through doping or vacancy processing, can affect the LSPR because of changes in the free electron density, the electron effective mass, and the electronic band structure in general [36–37]. An understanding of the changes in absorbance with respect to changing parameters of the material under consideration can be developed with a few examples of the theoretically arrived optical cross sections of a few generic morphologies of nanoparticles and will be discussed next for the case of nanospheres, nanorods, and nanomatryushkas.

The arrival at the expression for the LSPR frequency of a free electron cloud (as is typically assumed to be present in metals) starts with the relations between the dielectric displacement (*D*) of the electron gas in relation to the incident electric field (*E*) which it is [38] subjected to, given by


[1] $$D=\varepsilon_{0}E+P,$$

wherein *P* is the polarization density. *P* can be arrived at by solving the equation of motion for a single electron as


[2] $$P=\frac{-ne^{2}}{m\left(\omega^{2}+i\gamma \omega \right)E}.$$

Hence the expression relating the dielectric displacement (*D*) and the external electric field can be obtained as


[3] $$D=\varepsilon_{0}\left(1-\frac{\omega_{\text{p}}^{2}}{\omega^{2}+i\gamma \omega }\right)E,$$

where 
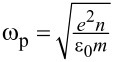
 is the natural frequency of oscillation of the electron cloud. Comparing Equation 3 to the general constitutive relation for a linear isotropic material given by Equation 4, we get the relation in Equation 5. ε_r_ is the relative permittivity of the material and ε_0_ the permittivity of free space.


[4] $$D=\varepsilon_{0}\varepsilon_{r}E,$$

[5] $$\varepsilon \left(\omega \right)=\left(1-\frac{\omega_{\text{p}}^{2}}{\omega^{2}+i\gamma \omega }\right).$$

For frequencies close to ω_p_, the temporal duration of damping (quantified by the product of ωτ, where τ is the relaxation time of the free electron gas) is much higher than unity, thus leading to an approximation that there is no damping. Hence ignoring the damping term in Equation 5, we get


[6] $$\varepsilon \left(\omega \right)=\left(1-\frac{\omega_{\text{p}}^{2}}{\omega^{2}}\right).$$

It follows also that under plasmon resonance conditions ε_1_ < −ε_m_, ε_m_ is the dielectric constant of the surrounding medium and hence the LSPR frequency can be arrived at as [39]:


[7] $$\omega_{\max}=\frac{\omega_{\text{p}}}{\sqrt{2\varepsilon_{\text{m}}+1}}.$$

Thus, for any morphology of a plasmonic nanoparticle, the LSPR frequency is intimately tied to the free electron density and the dielectric constant of the surrounding matrix. These factors thus decide the shape, position, and width of the plasmonic absorption and will be further elaborated in the subsequent sections.

**Figure 2:**
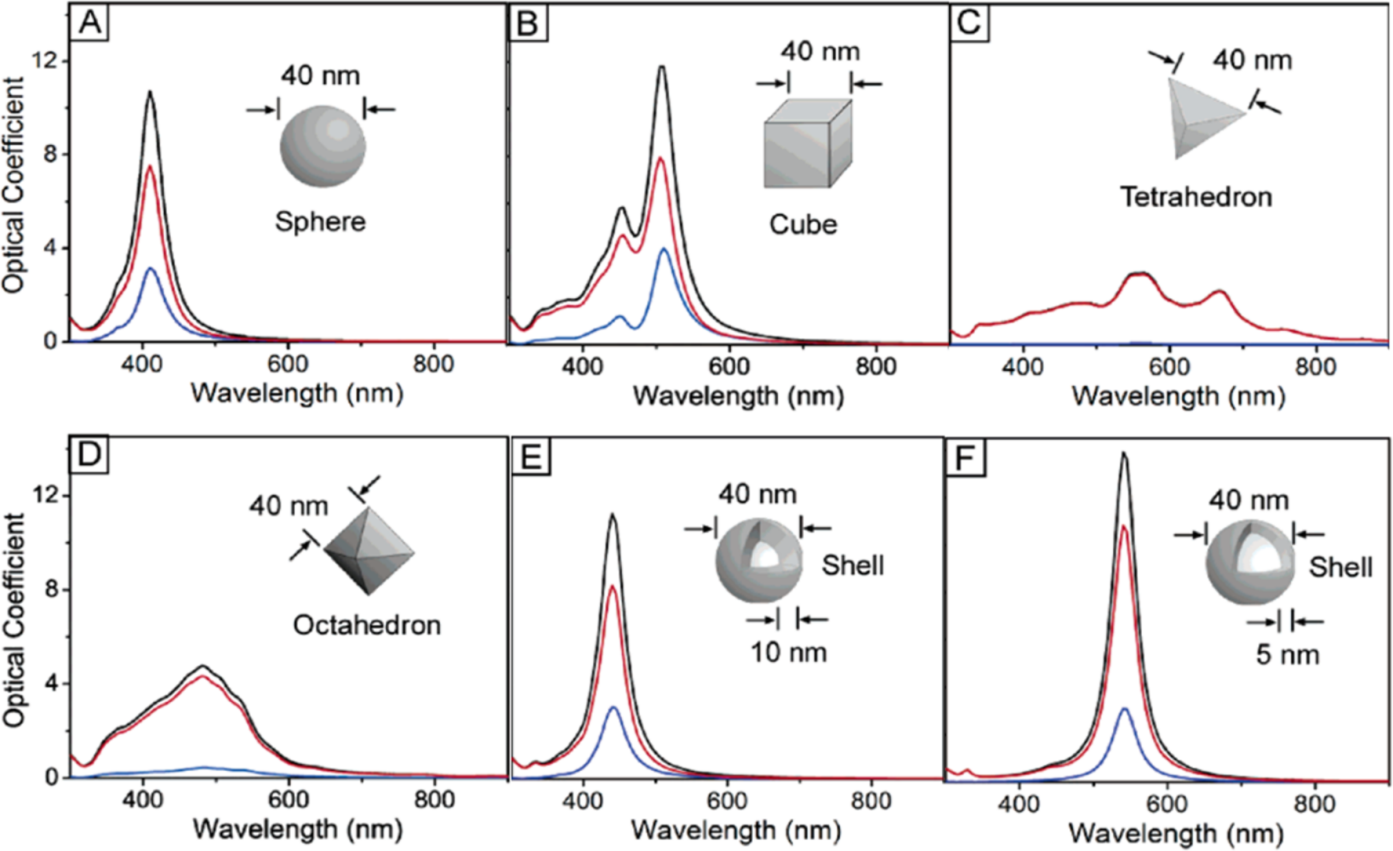
Optical spectra (absorption – red, scattering – blue, and extinction – black) of different morphologies of Ag nanoparticles representing shape effects of (a) nanosphere, (b) nanocube, (c) tetrahedron, (d) octahedron, and (e, f) core–shell structures with different shell thicknesses. Figure 2a–f was reprinted with permission from [40], Copyright 2006 American Chemical Society. This content is not subject to CC BY 4.0.

Plasmon absorption is also determined by the nanoparticle shape, which, although it does not appear in the plasmonic frequency equation, manifests as a shape/size factor in the calculations of the extinction spectra of nanostructures. Illustrative examples of the absorbance spectra for different morphologies of Ag nanoparticles are shown in Figure 2, elucidating the influence of the same. The extinction spectra (the summation of absorption and scattering spectra) from which the shape effects of different morphologies on plasmon excitation can be understood are hence crucial for assessing the PT properties of nanomaterials.

**2.1.1 Tunability of the plasmon frequency – changes to the dielectric constant.** In 1834, William Whewell coined the term dielectric [41]. In a dielectric material, positive charges are arranged in the direction of electric field and negative charges opposite to the field, causing a polarisation. The wavelength-dependent electric field and dipole moment determine the dielectric property of the material and also the plasmon absorption (by affecting the polarizability, as was shown in prior sections) [42].

The real (ε_r_) and imaginary part (ε_i_) of the dielectric constant relate to the refractive index (*n*) and extinction coefficient (*k*), respectively. Energy from a time-varying incident electric field is dissipated in part as heat, termed as dielectric loss. It can be envisioned that this dielectric loss is hence an important attribute to be considered for PT applications. The dielectric loss (δ) can be expressed as:


[8] $$\tan\delta =\frac{\varepsilon_{\text{i}}}{\varepsilon_{\text{r}}},$$

where ε_i_ is the imaginary part of the dielectric function, and ε_r_ is the real part of the dielectric function [43]. Polarization occurs for charge carriers in a number of orbitals or band, which then very often overlap, leading to multiple transitions even during plasmonic resonance. For example, metals such as Au, Ag, and Cu have d-band electrons close to the Fermi surface, and the polarization (*P*) in the presence of an electric field (i.e., the plasmon oscillation) has contributions from interband transitions as well. The dielectric function must account for this, and thereby the conventional definition given as [44]:


[9] $$\varepsilon \left(\omega \right)=1+P/\varepsilon_{0}E\varepsilon$$

changes (for metals at optical frequencies, for example) to


[10] $$\varepsilon \left(\omega \right)=1+\chi_{\infty }+\chi_{\text{D}}\left(\omega \right).$$

χ_∞_ is the susceptibility arising from the core electron polarizability (causing interband transitions), and χ_D_ is the corresponding susceptibility of the conduction electrons (modelled through the Drude assumption of a free electron “sea”). Hence, PT applications targeting plasmonic materials must account for the contributions to the dielectric function of the terms mentioned in Equation 10. A broad absorption of wavelengths is a reinforcing attribute of plasmonic materials for PT applications. However, increased broadening is associated with a reduced absorbance due to an increased scattering of the plasmon oscillations. An important phenomenon that broadens the plasmon line width is the scattering of plasmons at the surface for nanoparticles of sizes approaching the mean free path of electrons. Apart from the minor contribution to the linewidth arising from the disparity in particle sizes (when considering the absorption of a cluster of nanoparticles), the linewidth is controlled solely by surface scattering in such nanoparticles [45]. For free electron metals, the frequency of electron scattering (γ) is equal to the width of the plasmon resonance (Γ) [46]:


[11] Γ=γ.

In order to account for surface scattering, the linewidth needs to be modified as,


[12] $$\Gamma =\Gamma_{0}+\frac{A\nu_{\text{F}}}{a},$$

where ν_F_ is the Fermi velocity of electrons, Γ_0_ is the plasmon linewidth of the particles or damping constant, *a* is the radius of the spherical metal particles, and *A* is a parameter that depends on the scattering process. The final expression for the dielectric function taking into account the inter- and intraband transitions as well as the changed linewidth due to surface scattering (as appropriate), is


[13] $$\varepsilon \left(\omega \right)=\left(1-\frac{\omega_{\text{p}}^{2}}{\omega^{2}+i\omega \Gamma }\right)+\varepsilon {\left(\omega \right)}_{\text{inter}}.$$

Multiple inferences can be made from this formulation. In addition to linewidth broadening contributions to the dielectric function for smaller nanoparticles, electron orbit contractions (due to the majority of electrons being in proximity to the surface) result in an increased Coulombic force of restoration and hence a shift in the dielectric function [47]. Similarly, with a decrease in grain size, due to the fact that there is an increase in the volume fraction of grain boundaries compared to the grains, and since the dielectric strength of a grain is lower than a grain boundary, the dielectric permittivity decreases with decreasing grain size [48]. Moreover, the interaction between plasmonic nanoparticles and substrates on which they are deposited cannot be ignored. The polarization of charges in the nanoparticles induces dipoles in the substrate atoms in proximity of this polarization field, which in turn affects the nanoparticle resonance. This has been observed to induce higher-order resonances when the mismatch between the permittivity of the substrate and the surrounding of the nanoparticle increases, as well as to (depending on the orientation of the applied field with respect to the induced fields in the nanoparticle and the substrate) increase or decrease in the absorption intensity [49–51].

**Figure 3:**
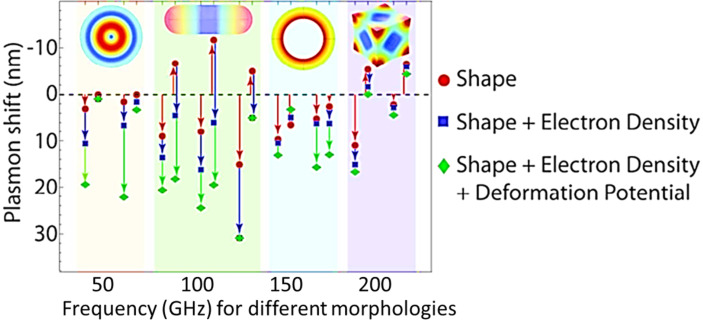
Manifestation of the governing factors of SPR. Shown are the changes to the peak position while considering only the shape (red circles), the shape as well as the electron density (blue squares), and the combination of shape, electron density, and deformation potential (green diamonds). Figure 3 was reprinted with permission from [53], Copyright 2017 American Chemical Society. This content is not subject to CC BY 4.0.

It follows from the discussion that considerable effects to the plasmon resonance are affected by the dielectric surrounding the plasmonic nanoparticle, apart from the permittivity of the nanoparticle itself. The shift in resonance on incorporation of the nanoparticle into a dielectric will decide its absorbance and hence the efficiency of conversion of the resonance into heat. This has been verified experimentally in multiple studies, wherein plasmon absorption peak shifts of up to 150 nm [52] have been observed with just a unit change in refractive index of the surrounding medium. As important as the dielectric function, the free electron density of the nanoparticle is also crucial for PT applications. This factor decides multiple attributes of a material, such as the quantum of this value that makes a given material plasmonic, the shifts in absorption and linewidth, and the ratio of radiative to non-radiative to radiative damping. A discussion on the manifestation of the free electron density on the plasmonic performance of different nanomaterials follows and is illustrated in Figure 3.

**2.1.2 Tunability of the plasmon oscillation frequency – changes to the free carrier density:** Free carrier densities of electrons are in the range of 10^22^ to 10^23^ cm^−3^ for plasmonic metals such as Au, Ag and Cu [54]. The free electron density is tied to the effective mass and determines the resonant plasma frequency (ω_p_), given by


[14] $$\omega_{\text{p}}^{2}=\frac{\pi Ne^{2}}{m},$$

where *N* is the free electron density, *m* is the effective mass, and *e* is the elementary charge. The LSPR effect is not present in most of the semiconductors because of their lack of the required free carrier concentration. Similar to how the free carrier density of metals can be tuned by size, morphology, and refractive index of the nanomaterial, the free carrier density of semiconductors can be easily tuned by doping, temperature variations, or by phase transitions. LSPR in semiconductors in the NIR–mid-IR region is possible when the free carrier concentrations lies between 10^16^ and 10^19^ cm^−3^ [54].

As an example of how the free electron density influences the plasmon resonance when materials with different work functions are combined, metals in contact with semiconductor metal oxide nanoparticles exhibit the spill-over effect, which alters the plasmonic absorption and spectral width of the plasmonic nanoparticles integrated in dielectric matrices. This spill-over effect, however, decreases with a decrease in electron density. For nanoparticles with low electron density (typically for radii less than 10 nm), the omnidirectional diffuse scattering dominates the resonance and the spill-over effect can be safely neglected. The spill-over effect and diffusive scattering can be related by [55]:


[15] Γspill-outΓdiffusive≅2.33m*rsIm[−dT(ωScl)]a0.

m* is the effective mass of an electron, *r*_s_ is the radius of on-electron, *d*_T_ is the complex length, 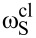 is the classical Mie LSPR frequency of the sphere = 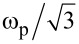, and the Bohr radius is 
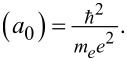


**Figure 4:**
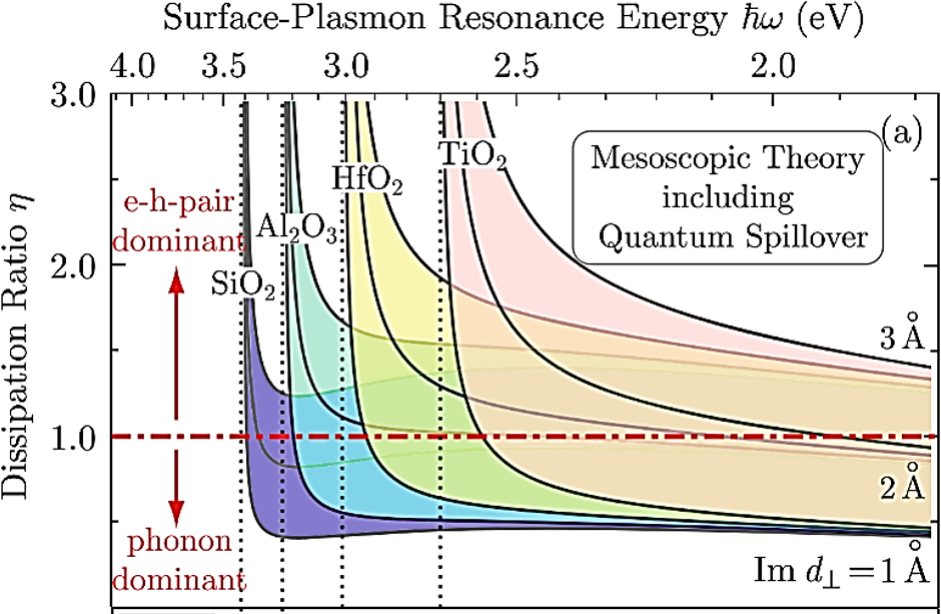
Dissipation ratio of electron–hole pair loss vs phonon loss of the surface plasmon excitation for different metal oxides. Figure 4 was reprinted with permission from [56], Copyright 2015 by the American Physical Society. This content is not subject to CC BY 4.0.

The free electron density can be tuned to make non-plasmonic materials plasmonic, which is useful for PT applications that require materials with properties that are not present in conventional plasmonic materials, such as a higher melting point, alloying capabilities, as well as possibly lower reactivities. Morphologies with optimal PT properties in terms of optical absorbance can benefit well from prior knowledge of the same, which can be obtained quite well through modelling efforts. Modelling of the absorbance of a plasmonic nanoparticle is done by calculation of its optical cross sections and specifically the extinction cross section, which includes the absorption as well as the scattering cross section. A few examples of the same will be discussed to compare the differences that need to be accounted for regarding different morphologies, essential for obtaining the accurate results in Figure 4.

#### 2.2 Extinction properties of nanomaterials

**2.2.1 Nanospheres:** The interaction of light with a particle is in one way simplistically modelled using the so-called quasi-static approximation where the incident electric field is assumed to be spatially uniform. This assumption is valid only for wavelengths much larger than the particle size. The extinction cross section, which is the result of this modelling and which is an expression summing up absorption and scattering of the incident radiation, is derived starting from Laplace’s equation with an electric potential (ϕ),


[16] $$\nabla^{2}\phi =0.$$

Calculation of the resulting scalar potentials inside and outside the particle leads to the expression for the polarizability (α) of the particle,


[17] $$\alpha =4\pi a^{3}\frac{\left(\varepsilon -\varepsilon_{\text{m}}\right)}{\left(\varepsilon +2\varepsilon_{\text{m}}\right)}.$$

ε_m_ is the dielectric constant of the surrounding medium. The extinction coefficient (σ_ext_) is introduced as:


[18] $$\sigma_{\text{ext(total)}}=\frac{1}{3}\left(\sigma_{\text{ext(}x\text{)}}+\sigma_{\text{ext(}y\text{)}}+\sigma_{\text{ext(}z\text{)}}\right),$$

[19] Cext=kIm(α),

where *C*_ext_ is the extinction cross section. On substituting for the polarizability, the final expression for the extinction cross section [57] for a spherical particle interacting with light is arrived at as


[20] $$Q_{\text{ext}}=\frac{24\pi a}{\lambda }\frac{\varepsilon_{2}\varepsilon_{\text{m}}}{{\left(\varepsilon_{1}+2\varepsilon_{\text{m}}\right)}^{2}+\varepsilon_{2}{}^{2}},$$

where *a* and ε_m_ are the particle size and dielectric constant of the surrounding medium, respectively, and ε_1_ and ε_2_ are the real and imaginary parts of the dielectric function of the material.

**Figure 5:**
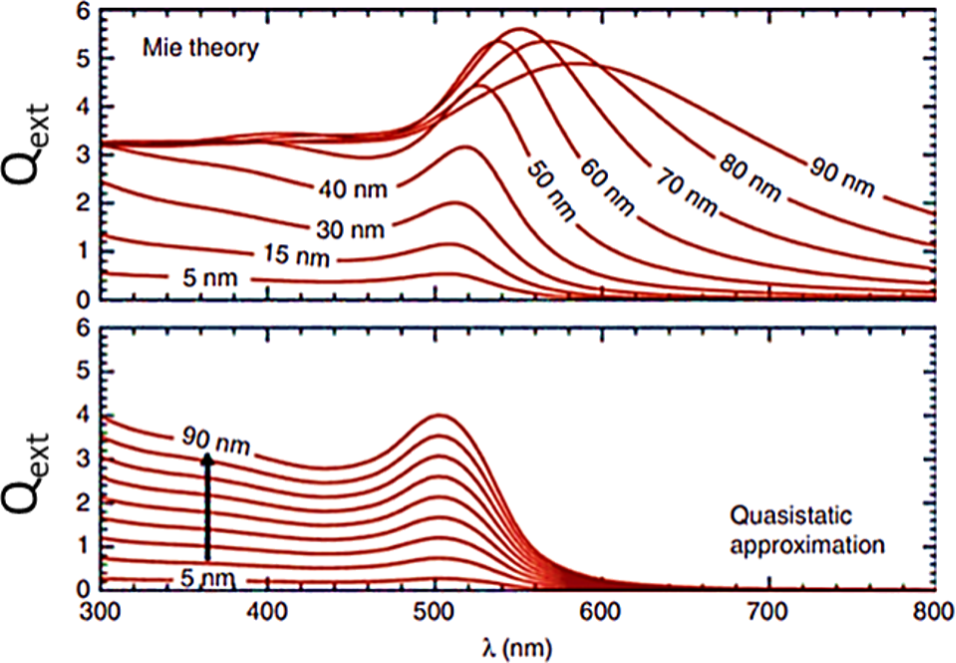
Extinction efficiencies of gold nanospheres calculated through the quasi-static approximation vs using Mie theory. The expected trends in the plasmonic peak shifts with particle size post the quasi-static limit can be seen to be predicted better by Mie theory. Figure 5 was reprinted by permission from Springer Nature from [58] ("Optical Properties of Metal Nanoparticles" by N. Harris et al., in Encyclopaedia of Nanotechnology, 2nd edition, Springer Dordrecht 2016, pp. 3027–3048), Copyright 2016 Springer Nature. This content is not subject to CC BY 4.0.

For larger particles (above approx. 40 nm), field-retardation effects affect the resonance position due to increasingly higher radiative damping, with significantly different extinction spectra arising also from the excitation of multipole resonances, which is not captured by the quasi-static approximation as shown in Figure 5.

The Mie theory solution of the extinction cross section is used to account for this through the inclusion of a size parameter (*x* = 2π*a*/λ) as well as through a coefficient for covering partial electric and magnetic waves (for the multipole orders). The extinction cross section is then given by


[21] σext=(2π/K2)∑j=1∞(2j+1)Re(aj+bj),

where


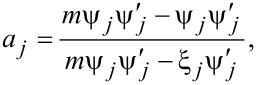



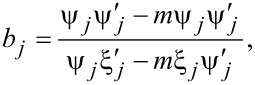



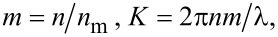



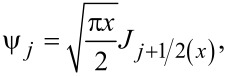



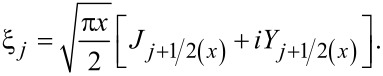


Here, ψ and ξ*_j_* are the Riccatti–Bessel functions, and *J* and *Y* are Bessel functions of the first and second order, respectively, *m* is the ratio of *n*_m_ and *n*, the real refractive index of the surrounding medium and the complex refractive index of the spherical nanoparticles, and *j* is an integer representing the order of scattering (dipole, quadrupole and so on). These equations for the extinction cross section more accurately capture the absorbance for particles larger than 40 nm. Even by accounting only for the dipole mode in such particles, depending on the order to which ψ*_j_* and ξ*_j_* are expanded, the shift as well as the changes to the peak broadening can be captured. For example, expanding the function to the order of *x*^2^ yields [58]:


[22] $$Q_{\text{ext}}=\frac{24\pi a}{\lambda }\frac{\varepsilon_{2}\varepsilon_{\text{m}}}{\left(\varepsilon_{1}+2\varepsilon_{\text{m}}+12x^{2}/5\right)+\varepsilon_{2}{}^{2}}.$$

**Figure 6:**
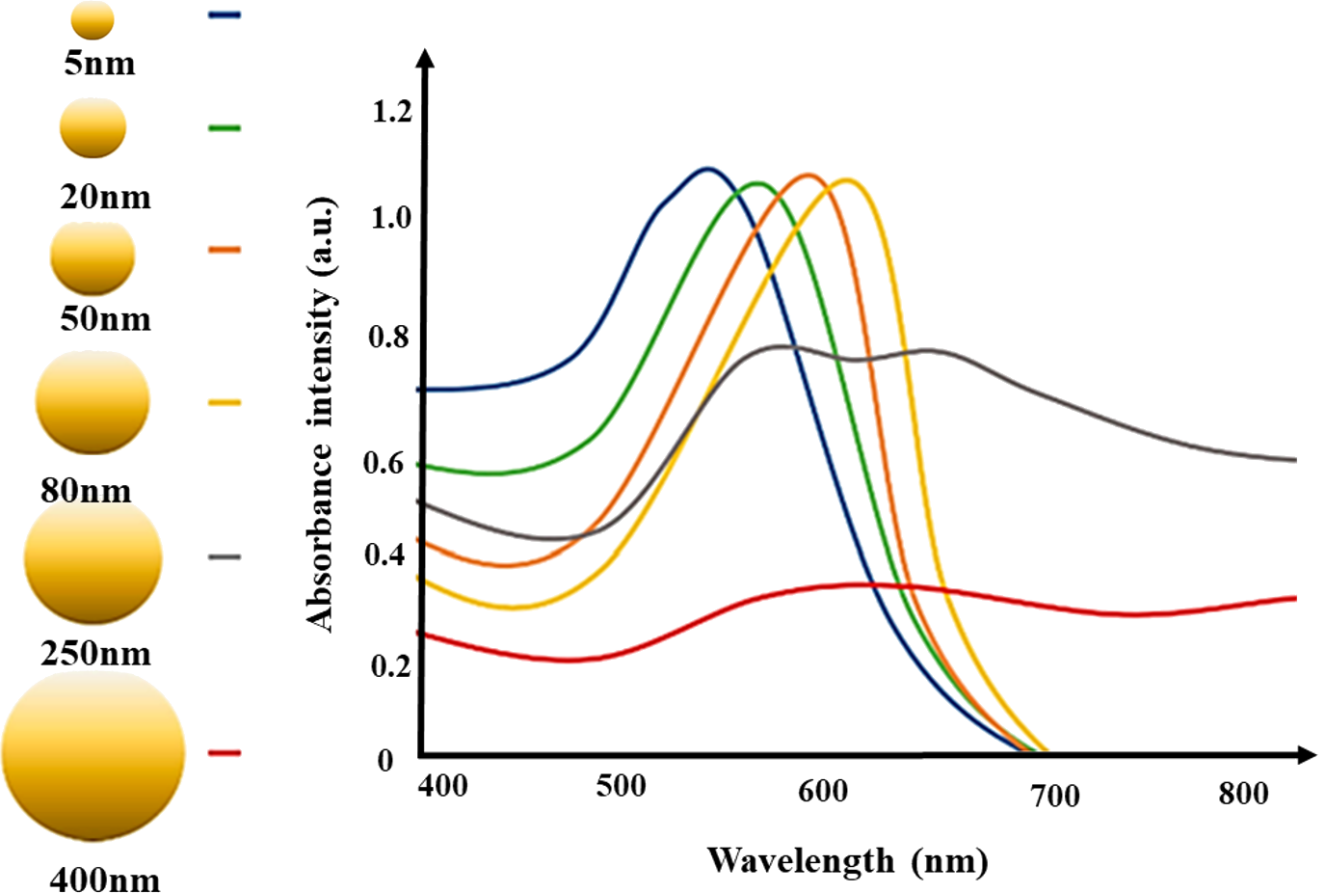
Absorption spectra of Au nanospheres of different diameters showing the shift of excitation wavelengths. The broadening of the SPR curve due to damping effects is evident. Figure 6 was reprinted from [59] (© 2018 H. S. Kim and Y. Lee, distributed under the terms of the Creative Commons Attribution 4.0 International License, https://creativecommons.org/licenses/by/4.0).

This equation captures well the redshift in the plasmon resonance for increasing particle diameters, not observable with the quasi-static approximation alone, as shown in Figure 6 for the case of Au nanospheres. Redshift and broadening of the plasmon resonance for the simple spherical morphology, allowing tunability of the absorption characteristics, are also evident from this discussion.

A common analogue to the spherical morphology are concentric nanoparticles, wherein a core nanoparticle is surrounded by a shell layer of specified thickness and composition. The change in refractive index of the shell in comparison to the core leads to a multitude of interesting effects such as altered/enhanced absorption and stability [60]. Hence core–shell nanoparticles can be tuned very effectively to the desired wavelength range by manipulating the thickness and/or composition of the shell in addition to the tunability prospects of the core itself, such as size and composition.

**2.2.2 Nanorods:** Nanorods in the shapes of cylinders or rectangles are a very common morphology explored in applications of sensing, catalysis, and plasmonics. Their attributes, including aspect ratio (ratio of the length to width), curvature, homogeneity, dimer formation, and placement, make them very interesting for multiple applications such as communication, hot-carrier enhanced catalysis, high-temperature sensing [61], and electronics applications such as transistors. Their extinction coefficient is often calculated using Gans theory for randomly oriented nanorods (with the rod geometry assumed as prolate spheroids with three principal axes) in the dipole approximation:


[23] $$\gamma =\frac{2NV\pi \varepsilon_{\text{m}}^{\frac{3}{2}}}{3\lambda }\sum_{j}\frac{\left(1/P_{j}{}^{2}\right)\varepsilon_{2}}{{\left(\varepsilon_{1}+\left(1-P_{j}\right)\frac{1}{P_{j}}\varepsilon_{\text{m}}\right)}^{2}+\varepsilon_{2}{}^{2}},$$

where ε_m_ is the dielectric constant of the surrounding medium, *P_j_* is the depolarization factor along the *A*, *B*, and *C* axes, and *R* is the aspect ratio of the nanorods (*B*/*A* or width/length). For example:


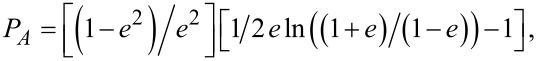



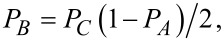



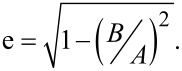


Because of the distinct and significant changes to the polarization of the electron cloud with respect to the elongation (aspect ratio) of the rods and any such rod-like nanostructures, considerable changes to the absorption properties are observed. This is because in addition to the polarization changes, there are also changes in the scattering processes (radiative vs non-radiative) of the plasmon oscillations. It follows that elongated nanostructures of plasmonic nanoparticles are more conducive for PT applications than spherical particles due to the precise and disparate manipulations possible in the absorbance of the former.

**2.2.3 Matryushka:** Multilayered nanoparticles have interestingly different optical characteristics than their single or non-layered pristine counterparts. Taking multiple forms such as core–shell, sandwich structures, and films, such multilayered nanostructures exhibit tunable plasmon resonance bands, resulting in additional shifts due to plasmon hybridization [62]. As an example, the interaction and hybridization of the plasmons of two separate metal shells (nanomatryushka) and two core dielectrics constitute the nanomatryushka (simplistically viewed as a ring within a ring). The coupling strength and energy between the plasmons on the inner and outer shells will determine the plasmon properties, which can be tuned by altering the dielectric spacer layer as well as the thickness of the specific nanoshells. Because of this intensified interaction between inner-core and outer-shell plasmons, PT energy transduction is significantly more effective.

The absorption coefficient (*C*_abs_) of the nanomatryushka (NM) can be calculated by varying the volumetric factor of the different layers of the nanostructures and the refractive index of the surrounding medium [63]:


[24] $$C_{\text{abs}}=2\int_{\text{NM}}P_{\text{LSPR}}\text{d}V/n_{\text{m}}\sqrt{\frac{\varepsilon_{0}}{\mu_{0}}}{\left|E_{\text{inc}}^{T}\right|}^{2}.$$

*P*_LSPR_ is the power absorbed by the nanoparticle, ε_0_ and μ_0_ are, respectively, the permittivity and permeability of vacuum, *n*_m_ is the refractive index of the surrounding medium, and 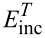 is the amplitude of the transverse incident wave of the laser source. Equation 24 can be simplified in the quasi-static limit as:


[25] CabsλLSPR=18πVNMλLSPRnm3Im{ε}

with


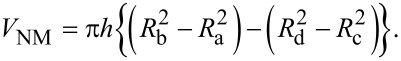


**Figure 7:**
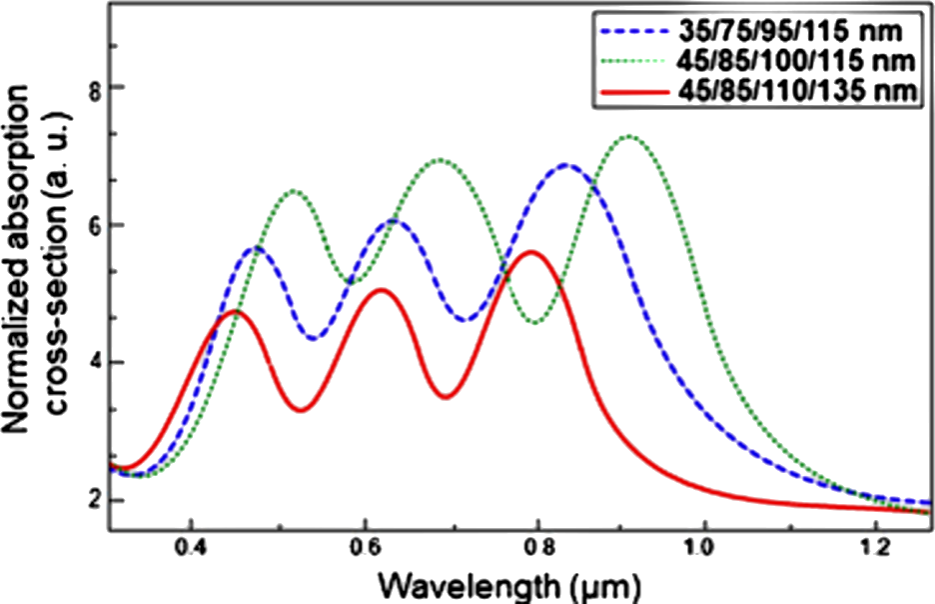
Normalized absorption spectra of Ag-NMs with different radii of the concentric spheres irradiated under with 1 mW/μm^2^. The appearance of periodic absorption peaks corresponding to multipolar excitations can be observed. Figure 7 was reprinted by permission from Springer Nature from [63] (“Analyzing Photothermal Heat Generation Efficiency in a Molecular Plasmonic Silver Nanomatryushka Dimer” by A. Ahmadivand; N. Pala, Plasmonics, Vol. 11, pp. 493–501, 2016), Copyright 2015 Springer Nature. This content is not subject to CC BY 4.0.

*h* is the height of the nanoshells, *R*_a_, *R*_b_, *R*_c_, and *R*_d_ are the radii of the different concentric spheres, λ_LSPR_ is the LSPR wavelength of the nanoparticle, and ε is the dielectric constant of the surrounding medium. In this nanomatryushka morphology, *h* represents the distance between the two concentric spheres, whereas in spherical nanoparticles the parameter inducing size effects was different. Hence, it is conclusive that the quasi-static approximation is valid for different morphologies but needs to account for the size when exceeding the validity of this approximation. Simulated absorption cross sections of Ag nanomatryushkas for different radii of different concentric spheres are shown in Figure 7.

From the few examples presented, it is clear that information such as the absorption range, the intensity of absorption, and importantly the changes in absorption resulting from changes to shape, size, material, and dielectric can be effectively obtained from simulations. Such endeavours minimize the effort required for material optimization. Also, they allow for considerable flexibility in exploring unconventional morphologies and material combinations. With continually improving computational resources, allowing for more complex calculations of extinction properties as well as PT conversion, modelling techniques are expected to play a symbiotic part with experiments in PT research. Understanding the processes that lead to the conversion of light to heat is critical for predicting and understanding PT performance, for which a discussion on the same is presented next.

#### 2.3 Relaxation mechanisms of plasmons – the conversion of photons to heat

When photons strike a plasmonic nanoparticle, plasmon excitations result in changes to the electron cloud thermal distribution. The electromagnetic absorptivity of the metal conduction electrons is proportional to the rate of momentum transfer to the lattice. It can be written as [64]:


[26] $$A\nu =\sqrt{\left(\frac{m^{*}}{4\pi e^{2}n}\right)}\frac{2}{\tau_{\text{eff}}}.$$

m^*^ is the effective electron mass, *n* is the electron density, and τ_eff_ is the conduction electron relaxation time given by


[27] 1τeff=25(ΘTτ).

**Figure 8:**
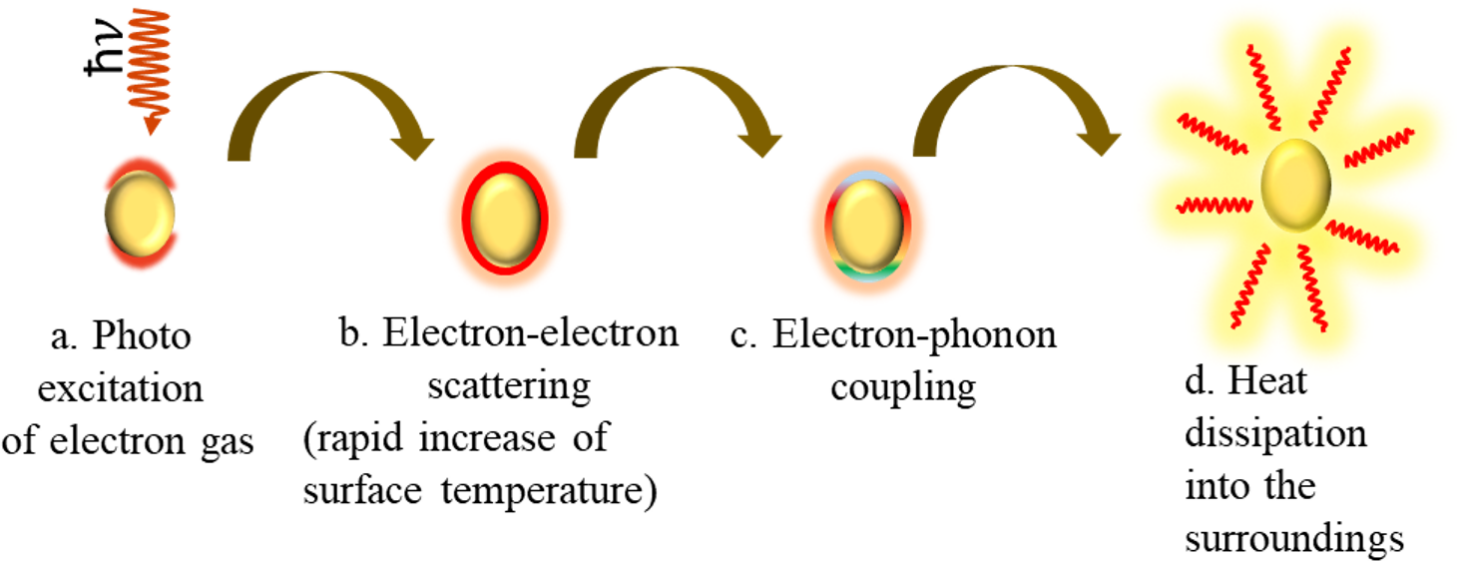
The PT conversion mechanism. (a) Photoexcitation of plasmons. (b) Electron thermalization. (c) Electron–phonon coupling. (d) Energy dissipation from the nanoparticle lattice to the surrounding. Figure 8a–d was redrawn from [66].

**Figure 9:**
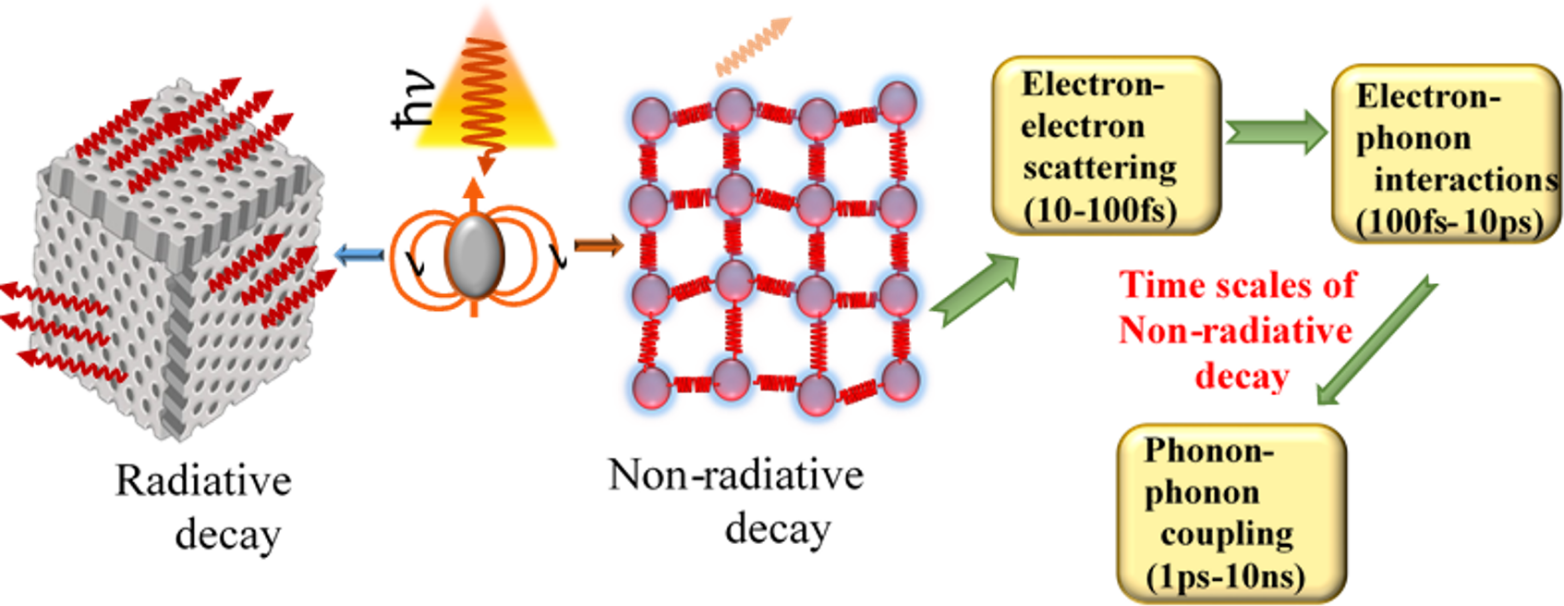
Radiative and non-radiative decay time scales of the conversion processes in PPT materials. For the right panel, the characteristic time scales of non-radiative decay were taken from [67].

τ is the conductivity relaxation time with temperatures *T* larger than the Debye temperature Θ. The Debye temperature is the temperature of a crystal’s highest mode of vibration. The decay of the excited electrons (plasmons) is through either radiative relaxation (i.e., photon emission) or non-radiative relaxation. Non-radiative relaxation occurs through the sequential processes of hot carrier generation, electron thermalization, and finally electron–phonon coupling heat transfer [65], leading to a temperature increase of the lattice (Figure 8 [66] and Figure 9).

Thus, the energy exchange between energetic plasmons and the phonon modes leads to an increase in temperature of the metal nanoparticles, and the ultimate conduction of this heat to the surrounding. The exchange time will vary for small nanoparticles due to quantum confinement effects [65] observed when the size of the nanoparticle is comparable to the Bohr exciton radius. In such cases, the confined electron wave functions have discrete energies and undergo considerably higher scattering with the surface because of the “shrunken” wave functions, contributing significantly to the reduction in absorption due to significantly increased linewidth broadening [68]. Interestingly, such small nanoparticles (typically smaller than 5 nm) have been found to undergo a much higher increase in temperature than larger nanoparticles (by more than five orders of magnitude) on irradiation with a single photon due to a much more efficient energy conversion [69–70]. Inferences from studies on PT relaxation in plasmonic nanomaterials are useful in designing them for such applications.

Electron–electron scattering can be understood to be the process following hot carrier generation due to plasmon relaxation, wherein thermalization of the electron distribution occurs. For a single scattering event, the duration of electron–electron thermalization is generally of the order of 10 fs (depending on the starting energy *E*) [52]. Accordingly, the influence of the initial energy on the electron–electron scattering time (τ_e–e_) with initial energy (*E*) and a Fermi energy (*E*_F_) can be expressed as


[28] $$\frac{1}{\tau_{\text{e}-\text{e}}}\propto {\left(E-E_{\text{F}}\right)}^{2}.$$

Electron–electron scattering is followed by electron–phonon coupling and heat transfer to the surrounding [71–72]. These non-radiative decay processes specifically concern PT applications.

**Figure 10:**
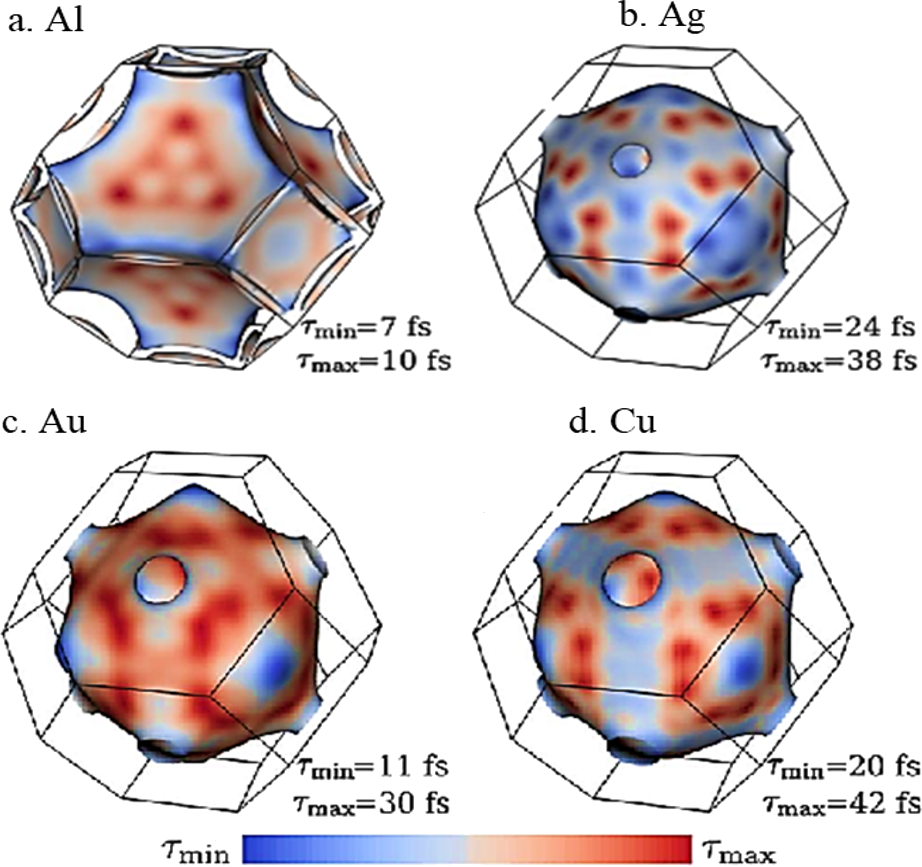
Hot carrier lifetimes on the Fermi surface and variation between positive and negative curvature, elucidating the variations occurring in relaxation rates and, by extension, electron thermalization of (a) Al, (b) Ag, (c) Au, and (d) Cu. Figure 10a–d was adapted with permission from [29], Copyright 2016 American Chemical Society. This content is not subject to CC BY 4.0.

**Figure 11:**
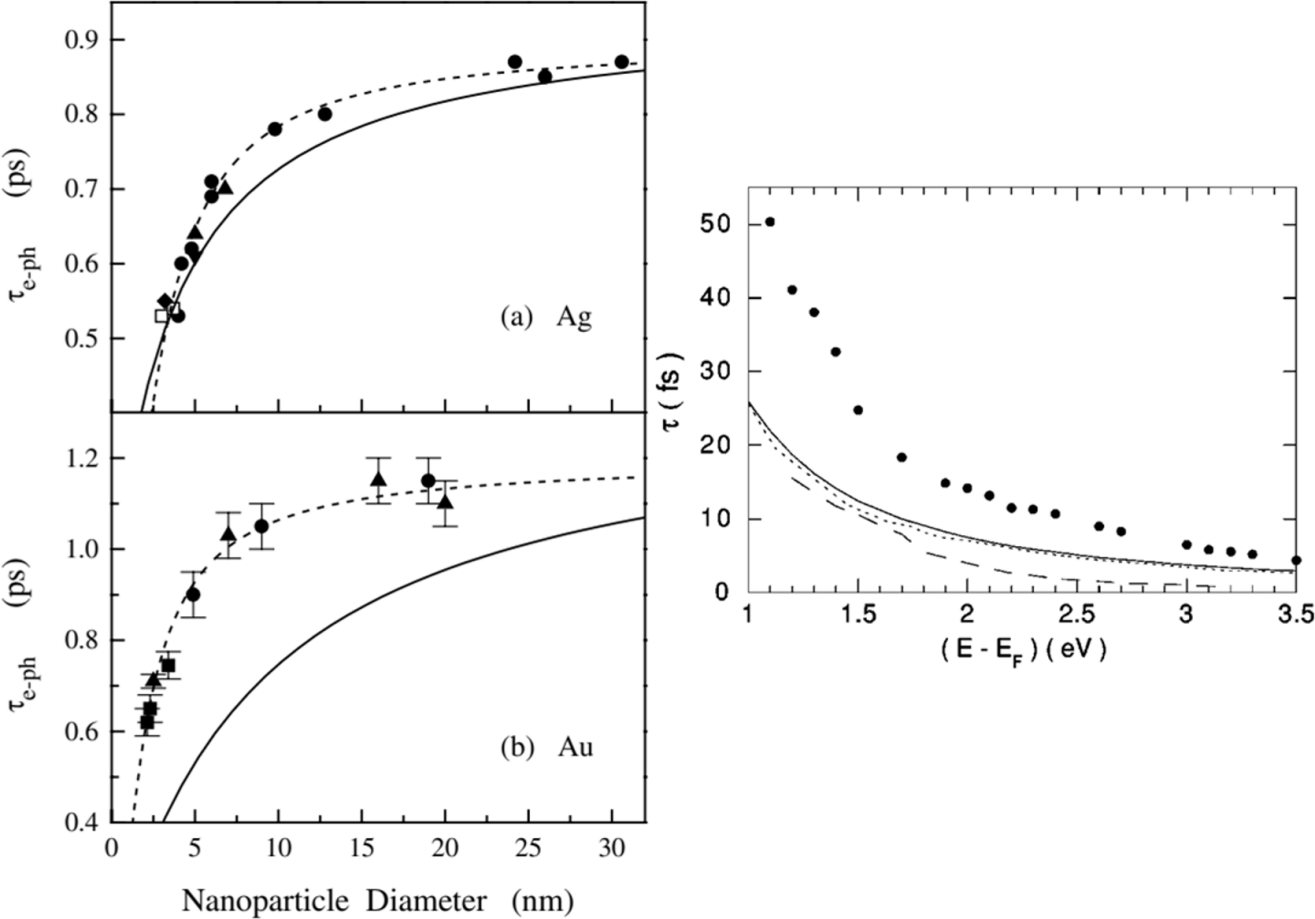
Electron–phonon coupling timescales for different diameters of metal nanoparticles: (a) Ag, (b) Au, and (right panel) Cu. Figure 11a and Figure 11b were reprinted with permission from [75], Copyright 2003 by the American Physical Society. This content is not subject to CC BY 4.0. Figure 11, right panel was reprinted with permission from [76] , Copyright 2000 by the American Physical Society. This content is not subject to CC BY 4.0.

Electron–phonon coupling, which reduces with decreasing particle size, and electron–surface scattering, which rises with decreasing particle size define the relaxation dynamics of the photoexcited electron gas into heat [52]. Interestingly, for Ag and Au, the transduction of absorbed light into heat due to electron–phonon coupling was found to be independent of the particle size above 10 nm diameter (though surface scattering effects start to dominate when the particle size is reduced beyond the mean free path of 40 nm for these nanoparticles) [73]. Electron–electron thermalization timescales for noble metals such as Au range from 10 to 100 fs, whereas the time scales for the electron–phonon interaction are slightly shorter, such as 10–30 fs for Al [74]. Hot carrier lifetime and electron–phonon coupling of selected metals are shown in Figure 10 and Figure 11. From the representation of electron filling for the different materials shown in Figure 10, it can be seen that the carrier lifetimes (depicted by the curvature of the Fermi surface) can vary considerably due to differences in the scattering rates of electrons that occupy the different energy levels.

The differences in the mean free path of carriers with the difference in their energy from the Fermi energy validates the observation that the higher the difference of the electron energy from the Fermi energy, the lower the mean free path. Therefore, it is essential for an understanding of the distribution of electrons based on their energy to predict the carrier lifetimes of plasmons, which in turn decides the damping properties of the resonance.

Since all mechanisms of plasmon relaxation (through non-radiative scattering) result in the eventual coupling of the scattered plasmons to phonons, their understanding is critical for designing materials for such applications. Simplistically, phonons are quantized particles or waves with a unit of vibrational energy corresponding to the continuum of frequencies ω(*q*) [77]. Their postulation and confirmation arose initially to reconcile deviations of the specific heat of materials at different temperatures from the Dulong–Petit law. The quantized characteristic (Eigen)energies of these vibrations are simply:


[29]

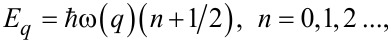



where ℏ is Planck’s constant, and *q* is the wave vector of the phonon oscillations. These elementary excitation phonons are bosons with a wave vector of *k* = 2π/λ. Crystal defects and other such sources of anharmonicity can change their frequencies and, hence, their coupling characteristics to electrons and the ultimate conversion of electron scattering into heat.

**Figure 12:**
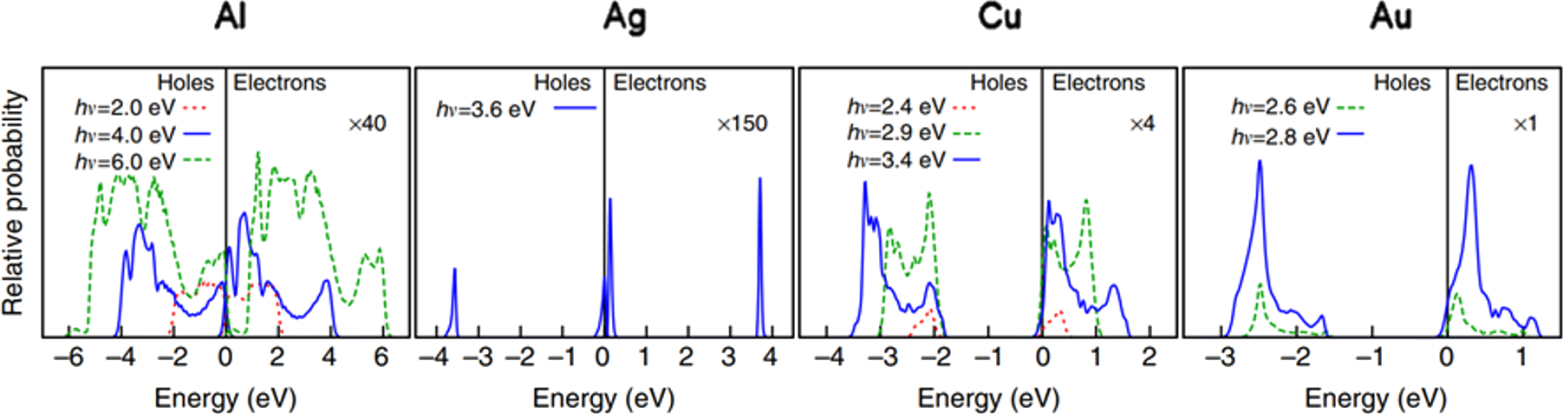
Comparison of the probability distribution of electrons and holes at various energy levels for coinage plasmonic metals. Figure 12 was reproduced from [80] (© 2014 R. Sundararaman et al., published by Springer Nature, distributed under the terms of the Creative Commons Attribution 4.0 International License, https://creativecommons.org/licenses/by/4.0).

The energy distribution of hot carriers (which decides the relaxation times) depends on the electronic band structure [78], particle size, density of states, and the geometry of nanoparticles [79]. Figure 12 shows the vast differences in the population of electrons and holes for Al, Ag, Au, and Cu. This distribution takes the characteristic of a symmetric/continuous distribution of electrons and holes in aluminium, a bimodal distribution in silver, and an antisymmetric hole-dominant distribution in copper and gold.

**Figure 13:**
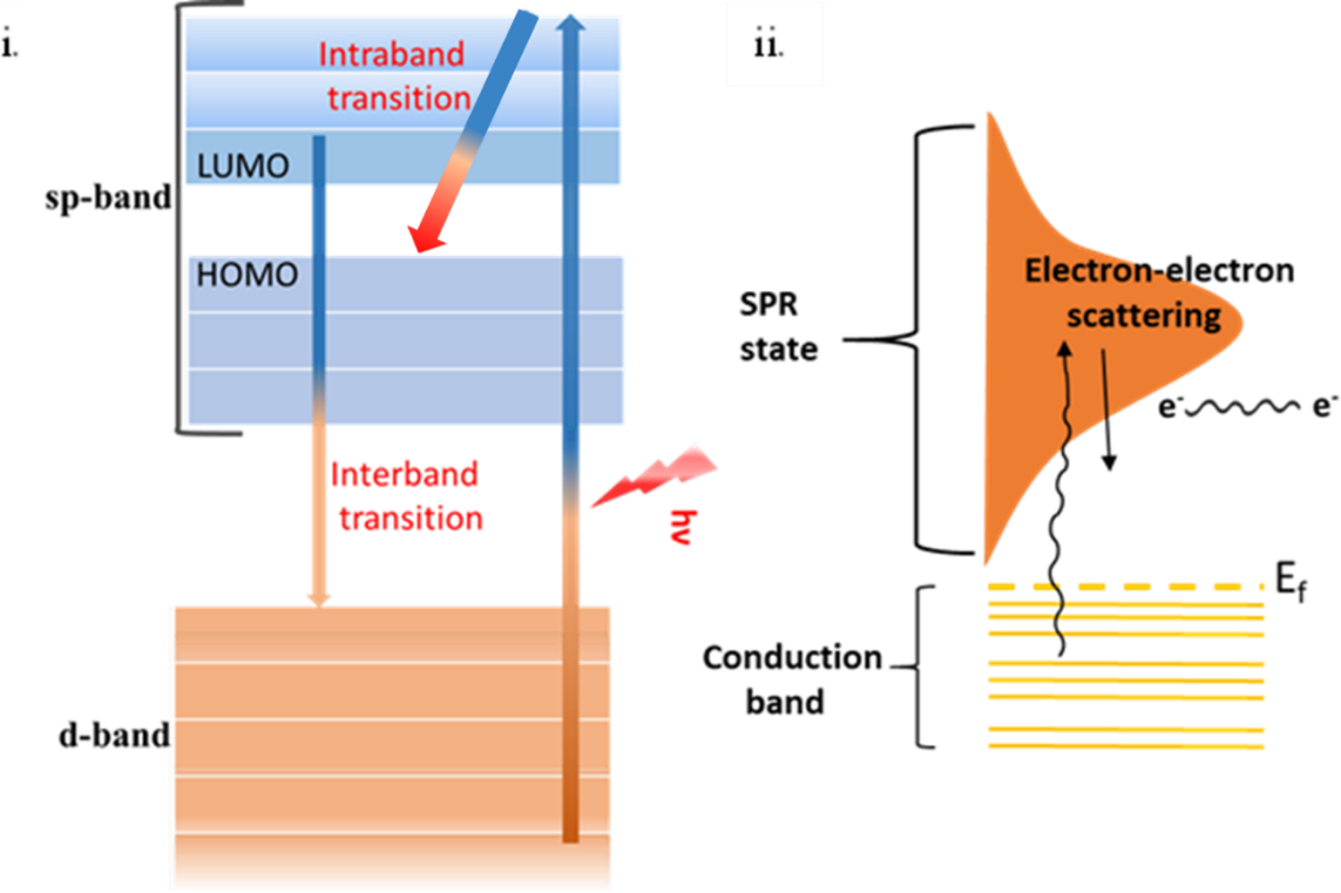
Relaxation process of the phonon vibration. Figure 13i (left panel) was redrawn from [82]. Figure 13ii (right panel) was adapted with permission of The Royal Society of Chemistry, from [72] (“Solar absorber material and system designs for photothermal water vaporization towards clean water and energy production”, by M. Gao et al., Energy & Environmental Science, vol. 20, issue 3, © 2019); permission conveyed through Copyright Clearance Center, Inc. This content is not subject to CC BY 4.0.

Also, decreasing the thickness of Au films from 40 nm increases hot carrier generation through increased intraband transitions as well as due to an asymmetric density of states distribution. Hence, hot carrier generation and lifetimes depend on the material, particle size and shape, as well as the distribution of the density of states [81]. Figure 13 illustrates the relaxation process of the phonon vibration for a better understanding of phonon dynamics.

The electron–phonon coupling constant, which describes the potential of a material for undergoing lattice heating, is calculated by transient reflectivity studies, for example through pump–probe laser spectroscopy [83]. From the slope of this transient reflectivity curve, the coupling constant is arrived at, from which the lattice temperature is calculated through the two-temperature model by means of two coupled diffusion equations,


[30] $$\frac{\text{d}T_{\text{e}}}{\text{d}t}=\frac{-g\left(T_{\text{e}}-T_{\text{1}}\right)}{C_{\text{e}}\left(T_{\text{e}}\right)},$$

[31] $$\frac{\text{d}T_{1}}{\text{d}t}=\frac{g\left(T_{\text{e}}-T_{\text{1}}\right)}{C_{1}-\frac{\left(T_{1}-T_{0}\right)}{\tau_{s}}},$$

where *T*_e_ and *T*_l_ are, respectively, the electronic and lattice temperature, *C*_e_ and *C*_l_ are, respectively, the electronic and lattice heat capacities, *g* is the electron–phonon coupling constant, and *T*_0_ is the ambient temperature.

It can be seen that the governing fundamental particles in PPT heating are phonons, and that the tailoring of PT heating properties by phonons can thus be effectively realized by lattice tuning of the pristine materials, that is, by defect tailoring, doping, or by providing external stimuli such as pressure.

While considering the actual increases in temperature of the nanoparticle possible due to plasmonic heating, the differences in specific heat capacity between the lattice and an electron must be considered. Electrons having a much smaller specific heat capacity compared to the lattice will undergo much higher heating than the lattice as a whole. For example, for differences in the electron temperature (due to the initial thermalization step) up to 1000 °C, the increase in nanoparticle temperature was found to be only 20 °C [84]. This implies by no means a weak efficiency of heat production due to plasmon relaxation, as high temperatures (230 °C for AgNPs for example) are possible, depending on the various factors that have been discussed in preceding sections [85]. PPT heating has subwavelength spatial selectivity, implying that changes to absorption and, hence, heating can be affected by means of changing the lasing power density and/or the wavelength. Indeed, NP morphologies have even been shown to be controlled due to extremely localised heating [38].

For increasing the spatial distribution of the generated heat, compared to an individual nanoparticle, nanoparticle assemblies and/or increased interfaces (by including constructions such as holes and other scattering centres) within a single nanostructure can be effective [86–89]. A few governing equations which allow to predict the different aspects of PPT energy conversion are discussed next. They can be of use to explicitly select a material/morphology/matrix based on the application it is being considered for.

### 3 Calculations in PT heating

Calculations of PT heating metrics, such as the specific heat capacity of materials with added nanoparticles, the conversion efficiency, the rate of heat generated per volume, and the temperatures at different locations across the thermal front require appropriate equations. The PT conversion efficiency for water vaporisation can be calculated from


[32] $$\eta =\frac{\Delta m\Delta_{{\text{vapH}}_{\text{m}}}}{\text{MIS}\tau }.$$

Δ*m* is the water mass loss during irradiation, 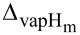 is the phase change enthalpy of water to vapour, *M* is the molar mass of water, *I* is the solar power intensity at the surface of the sample, *S* is the irradiated area of the water surface, and τ is the irradiation time. This equation is applicable for the case of nanoparticles dispersed in liquids for vapour generation, the most common non-medical application of PT energy conversion [90]. The equation governing the temperature distribution in an isotropic nanomaterial is given by


[33]

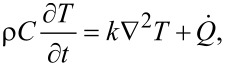



where ρ is the density, *C* is the specific heat, *k* is the thermal conductivity, *T* is the temperature, *t* is the time, and 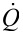 is the rate of heat generation (or depletion) per unit volume. The Laplacian ∇^2^ represents the temporal change in the temperature variation of the material. The rate of heat generation or depletion, 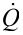, is arrived at from [91]:


[34]

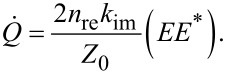



Solving the second-order Equation 33 requires specification of boundary conditions as well as initial conditions such as the type of heat transfer applicable to the specific study (conduction/convection/radiation), the geometry (radii, lengths, and similar dimensionally defined factors), direction of heat transfer, and the heat transfer coefficients for the material as well as the surrounding medium [84].

As the method of laser irradiance is generally employed in PT studies, to measure the temperature rise of the nanoparticles when irradiated with a laser source, the energy balance equation can be used [92–93]:


[35] $$\sum_{i}m_{i}C_{i}\frac{\text{d}T}{\text{d}t}=Q_{\text{in}}-Q_{\text{out}}.$$

*m_i_* and *C_i_* are, respectively, the mass and the specific heat capacity of the *i*-th component, *T* and *t* are, respectively, temperature and time, and *Q*_in_ and *Q*_out_ are, respectively, the rate of input power from the laser and the rate of heat dissipation to the surrounding. Often, nanomaterials are used in conjunction with a phase-change material for energy storage applications, and when plasmonic nanoparticles are integrated into a solid phase-change material (n-PCM), the energy balance equation is be given by [94]:


[36] $$\Delta H_{\text{fus}}\rho_{s}\left(1-\phi \right)\frac{\text{d}\delta {}^{\circ}}{\text{d}t}=\frac{T_{\text{m}}-T_{\text{c}}}{\frac{1}{h_{\text{c}}}+\frac{W}{k_{\text{w}}}+\frac{\delta }{k^{\prime }}}.$$

Δ*H*_fus_ is the heat of fusion, ρ_s_ is the density of solid, ϕ is the volume fraction of the particles, *T*_m_ is the melting temperature, *T*_c_ is the temperature of the cooling fluid, *W* is the thickness of the compartment holding the n-PCM, *k*_w_ is the conductivity of the wall, and k′ is the conductivity of the PCM. The thermal conductivity of the plasmonically enhanced PCM materials can be varied with the volumetric fraction of the PCM material and can be specified by [95]:


[37] $$\begin{array}{c} K_{\text{nPCM}}=\frac{K_{\text{np}}+2K_{\text{PCM}}-2\left(K_{\text{PCM}}-K_{\text{np}}\right)\phi }{K_{\text{np}}+2K_{\text{PCM}}+\left(K_{\text{PCM}}-K_{\text{np}}\right)\phi }K_{\text{PCM}}\\ +5\times 10^{4}\beta_{\text{k}}c\phi \rho_{\text{PCM}}C_{\text{pPCM}}\sqrt{\frac{k_{\text{B}}T}{\rho_{\text{np}}d_{\text{np}}}}f\left(T,\phi \right). \end{array}$$

*k*_B_ is the Boltzmann constant, β_k_ = 8.4407 × (100ϕ)^−1.07304^, *f*(*T*,ϕ) = (2.8217 × 10^−2^ϕ + 3.917 × 10^−3^) *T*/*T*_ref_ + (−0.669 × 10^−2^ϕ − 3.91123 × 10^−2^), *K*_np_ and *K*_PCM_ are the thermal conductivities of nanoparticles and PCM, respectively, ϕ is the volumetric fraction of the nanoparticles, ρ_np_ and ρ_PCM_ are the densities of nanoparticle and PCM, respectively, and *C*_pPCM_ is the specific heat capacity of the PCM.

The thermal capacitance coefficient relates the rise in temperature of the irradiated nanoparticles to the thermal conductivity and absorption power of the surrounding medium. The temperature increase in the metal nanoparticles (Δ*T*_NP_) and thermal capacitance coefficient can be calculated using the following equation [96]:


[38] $$\Delta T_{\text{NP}}=\frac{I\sigma_{\text{abs}}}{4\pi \beta Rk_{\text{med}}}.$$

**Figure 14:**
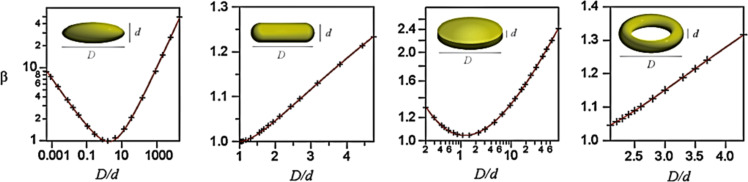
Thermal capacitance coefficient (β) for the ellipsoidal, rod, disk, and ring morphologies of Au nanoparticles as a function of the aspect ratio *D*/*d*. Figure 14 was reprinted with permission from [96], Copyright 2010 American Chemical Society. This content is not subject to CC BY 4.0.

*I* is the illumination intensity of the laser source, σ_abs_ is the absorption cross section of the nanoparticles for the relevant wavelength, β is the thermal capacitance coefficient, *R* is the radius of the sphere, and *k*_med_ the effective permittivity of the homogeneous medium. The thermal capacitance coefficients (*C*_th_) of the different morphologies mentioned in Figure 14 can be calculated as:


[39] $$C_{\text{th}}=4\pi R\beta k_{\text{med}}.$$

Employing the appropriate of the described equations ensures compatibility of assumptions and the observed results. Any deviations could imply a multitude of factors, such as differences in assumptions in terms of the dominant mechanism of heat transfer, a deficient representation of the particle geometry, or inaccuracies in modelling the temporal evolution of the generated heat, to name a few. With this understanding of the myriad of theoretical and phenomenological phenomena, considerations, and evaluations in PPT heating, the different materials that have been investigated are well worth reviewing to appreciate the intricacies in this field. This will be done while keeping in mind two important factors. One will be the different materials that are involved in PT heating, and the other will be the stability requirements for the materials used and possible means to improve this stability.

### 4 Plasmonically active nanomaterials for PT applications

#### 4.1 PT performance of metal nanoparticles

Metallic nanoparticles are a dominant group of elements for PT research. The calculations of different optical cross sections, namely absorption, scattering, and extinction allow for the prediction of the ideal candidate for maximizing PT efficiency. For example, in terms of maximizing the absorption and, hence, the interaction with solar radiation of these nanoparticles for steam generation, modelling of these cross sections with water as the surrounding medium can give precise results [97]. The ratio between absorption and scattering cross sections can be intuitively concluded to be a convenient measure of the absorption efficiency, as maximizing absorption whilst minimizing scattering implies a higher conversion of light into heat rather than re-emission as light. Considering the broad spectrum of metallic elements, nanospheres of gold, silver, platinum, cobalt, zinc, nickel, titanium, copper, aluminium, molybdenum, vanadium, and palladium have been analysed [98]. Although there were assumptions of homogeneity, sphericity, and no interaction between particles, the Mie theory-based scattering cross sections in this study can still be important for the assessment of optical properties. In this study the conclusion that all particles become increasing scatterers with size (from 10 to 50 nm), with an analogous trend in the maximal temperature increase of the nanoparticles, was made. The larger nanospheres (larger than 50 nm) acted more as absorbers than as scatterers for wavelengths of 200–500 nm for Au, 400–1000 nm for Pt, 600–1000 nm for Pd, Co, and Zn, 500–1000 nm for Mo, 200–600 nm for Cu, 300–1000 nm for Ni, 450–1000 nm for V, and 200–1000 nm for Ti. Al and Ag were inefficient absorbers and predominantly scattered the light for radii between 200 and 1200 nm. A detailed investigation on Au, Ag, Cu, and Al with regard to solar heating has been done wherein the effects of changing size and morphology of the nanoparticles and the dielectric environment were studied. An intuitive but important factor for selecting PT materials is the ratio between the absorption and scattering sections, termed the absorption ratio (α) and given by:


[40] $$\alpha =\frac{P_{\text{abs}}}{P_{\text{abs}}+P_{\text{scat}}}\times 100,$$

[41] $$P_{\text{abs}}=\int_{\lambda_{\min}}^{\lambda_{\max}}I\left(\lambda \right)C_{\text{abs}}\left(\lambda \right)\text{d}\lambda ,$$

[42] $$P_{\text{scat}}=\int_{\lambda_{\min}}^{\lambda_{\max}}I\left(\lambda \right)C_{\text{scat}}\left(\lambda \right)\text{d}\lambda .$$

*I*(λ) is the intensity of the incident radiation, *C*_abs_(λ) is the absorption cross section, *C*_scat_(λ) is the scattering cross section, and λ_max_ and λ_min_ are the maximum and minimum wavelength of the calculated solar radiation, respectively. The higher the ratio, the more suitable the material is for PT heating. With this in mind, a few of the key insights are the highly intense but narrow absorption cross section [99] of Au compared to other metals, the higher δ of Al and Cu, the higher temperature increases possible with reduced sphericity, the better absorption properties when surrounded by materials with a higher refractive index (due to multipolar excitations instead of only dipolar excitations), and the threshold particle size above which the optical scattering starts to increasingly manifest, reducing the PT heating efficiency (e.g., 30 nm spheres of Au). Such calculations can help in choosing the right plasmonic metal for an application, and since PT applications rely on maximal absorption by the plasmonic resonance, information on the required wavelength range and the available particle sizes can narrow down the choices.

**Figure 15:**
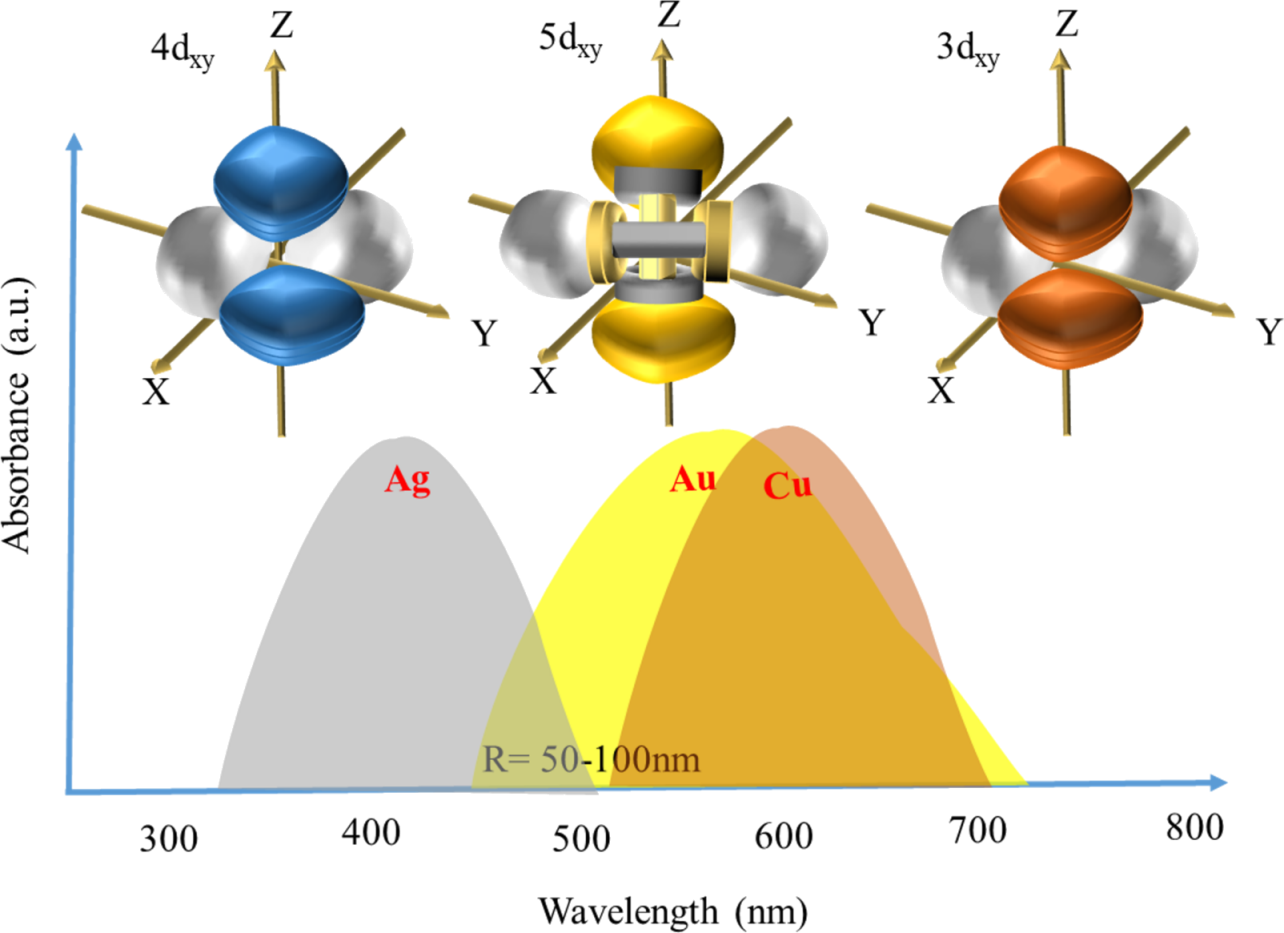
Absorption spectra of spherical nanoparticles of Ag, Au, and Cu with radii between 50 and 100 nm and the corresponding valence orbitals.

Metal nanoparticles in general are very efficiency in generating plasmons since they have many mobile electrons with a mean free path of 10 to 100 nm [100]. They are exceptional in their tunability of wavelength absorption due to tunability of shape, from 0-dimensional to 3-dimensional nanoparticles, size, and composition. The coinage metals Ag and Au have been explored traditionally for PT applications. A schematic representation of the plasmonic absorbance regimes of Au, Ag, and Cu for radii between 50 and 100 nm is shown in Figure 15. Au nanoparticles have been extensively used for PT applications because of the possible intricate control of morphology (and hence absorbance), the chemical stability, and the obvious plasmon-enhanced heat transfer that does not occur in low-cost, yet ineffective, non-plasmonic materials such as graphite and CNTs [101–103].

**Figure 16:**
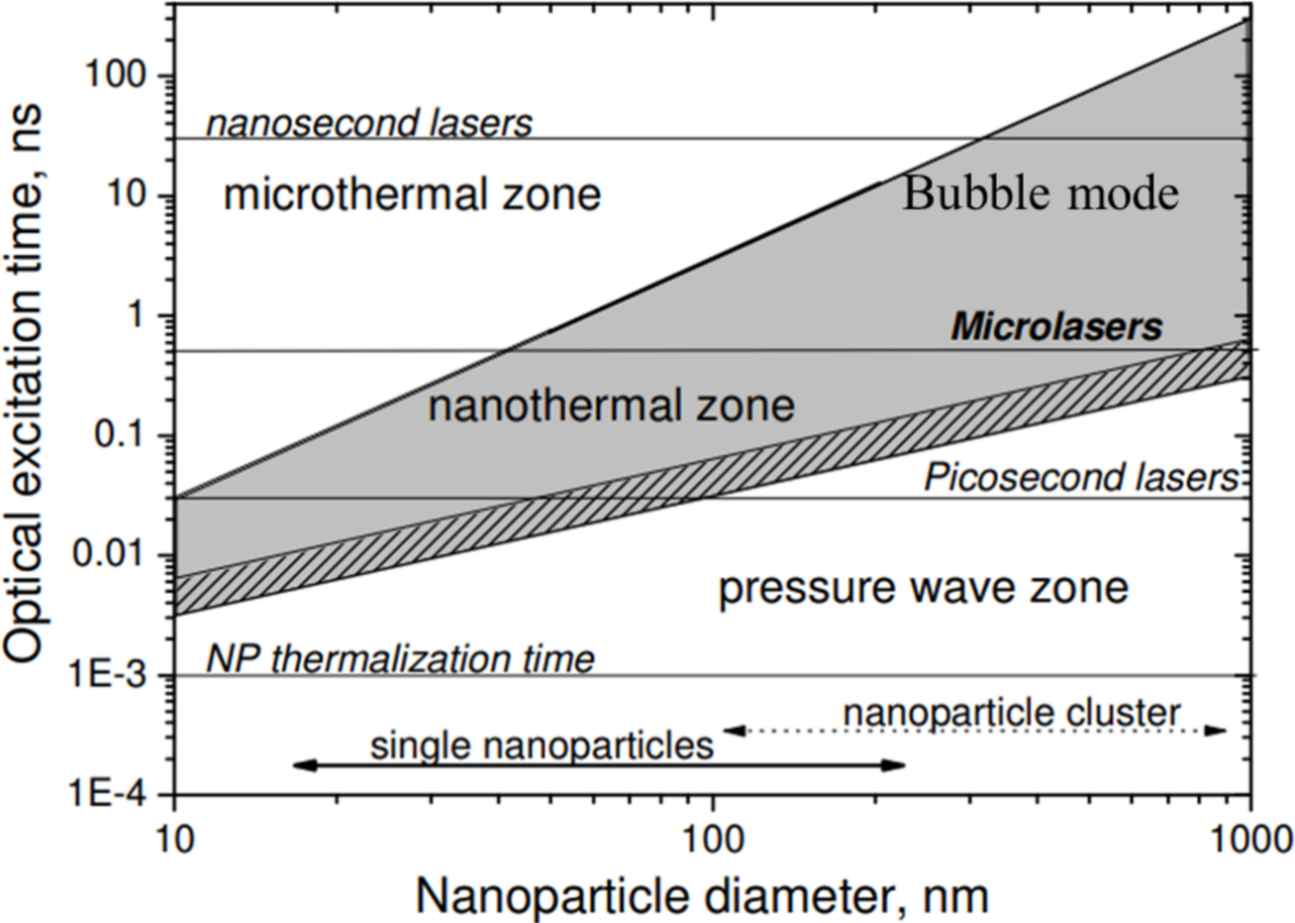
PT conversion and thermalization of plasmonic nanoparticles after absorption of a laser pulse. The hatched area shows the border between bubble mode and pressure wave mode due to the increase of the nanoparticle temperature during plasmonic interaction. Figure 16 was reprinted with permission from [106] (D. Lapotko, “Optical excitation and detection of vapor bubbles around plasmonic nanoparticles”, Opt. Express, vol. 17, issue 4, article no. 2538, 2009), © The Optical Society. This content is not subject to CC BY 4.0.

Excellent reviews on properties relevant to PT heating such as the relaxation dynamics, field distributions during SPR, shape and volume effects, and the optimal configuration of Au and Au-based nanomaterials in PT heating systems further support this view [87,104]. Careful studies done on plasmonic Au nanoparticles revealed the different mechanisms that can lead to vapour formation in steam generation. Particularly, the influence of the irradiance intensity on two contrasting mechanisms, namely explosive boiling and plasmonic nanobubble formation has been revealed [83]. Plasmonic nanobubbles (formed when the irradiation fluence exceeded a threshold value), although being excellent tunable scatterers themselves, did not result in thermal phenomena such as heating and only led to mechanical phenomena such as cavitation effects. Explosive boiling is of explicit use in PT applications, while its use in a novel synthesis strategy for SERS-enhancing self-assembled nanoparticles is worth mentioning [105]. Though details about the absence of thermal phenomena for plasmonic nanobubble formation are yet to be confirmed, the important conclusion that increasing the irradiation fluence will not necessarily lead to better PT properties can be made from such studies. This can be understood from a very helpful phase map (Figure 16) [106] showing the different phenomena that accompany the laser irradiation of plasmonic nanoparticles. The influence of irradiation fluence, pulse duration, continuous vs pulsed irradiation, morphology, and solvent has been studied in detail for Au nanoparticles regarding applications such as catalysis, bio-activation, steam generation, and thermoplasmonics.

For PT applications, polygonal Au structures are preferred over nanospheres because absorption wavelengths can be tailored and broadband absorbance is possible, in contrast compared to a single narrow absorbance peak for nanospheres as discussed in preceding sections. A ubiquitous example of such polygonal morphologies, the Au nanorod, has been investigated quite well with regard to the effect of the aspect ratio as well as different encapsulating shell materials (Ag_2_S and ZnS) on PT performance. The temperature increase depends on the difference between the exciting wavelength and the plasmonic wavelength, with minimal differences contributing to maximal temperature increase. An associated increase in the scattering cross section calculated theoretically validated this observation as scattering increases when the difference between the exciting wavelength and the plasmon wavelength increases. This difference can be reduced by shifting the resonance wavelength closer to the excitation wavelength, possible through incorporating a shell that can cause this shift due to changes in the medium refractive index, as can be understood from Equation 22. Shell materials can also increase PT efficiency by absorbing radiation over a broader range inaccessible by the plasmonic core [89]. The separation between excitation and resonance wavelength can also be reduced by inducing the assembly of isolated nanoparticles, such as the assembly of Au rods and polyhedral particles in the presence of glutathione.

**Figure 17:**
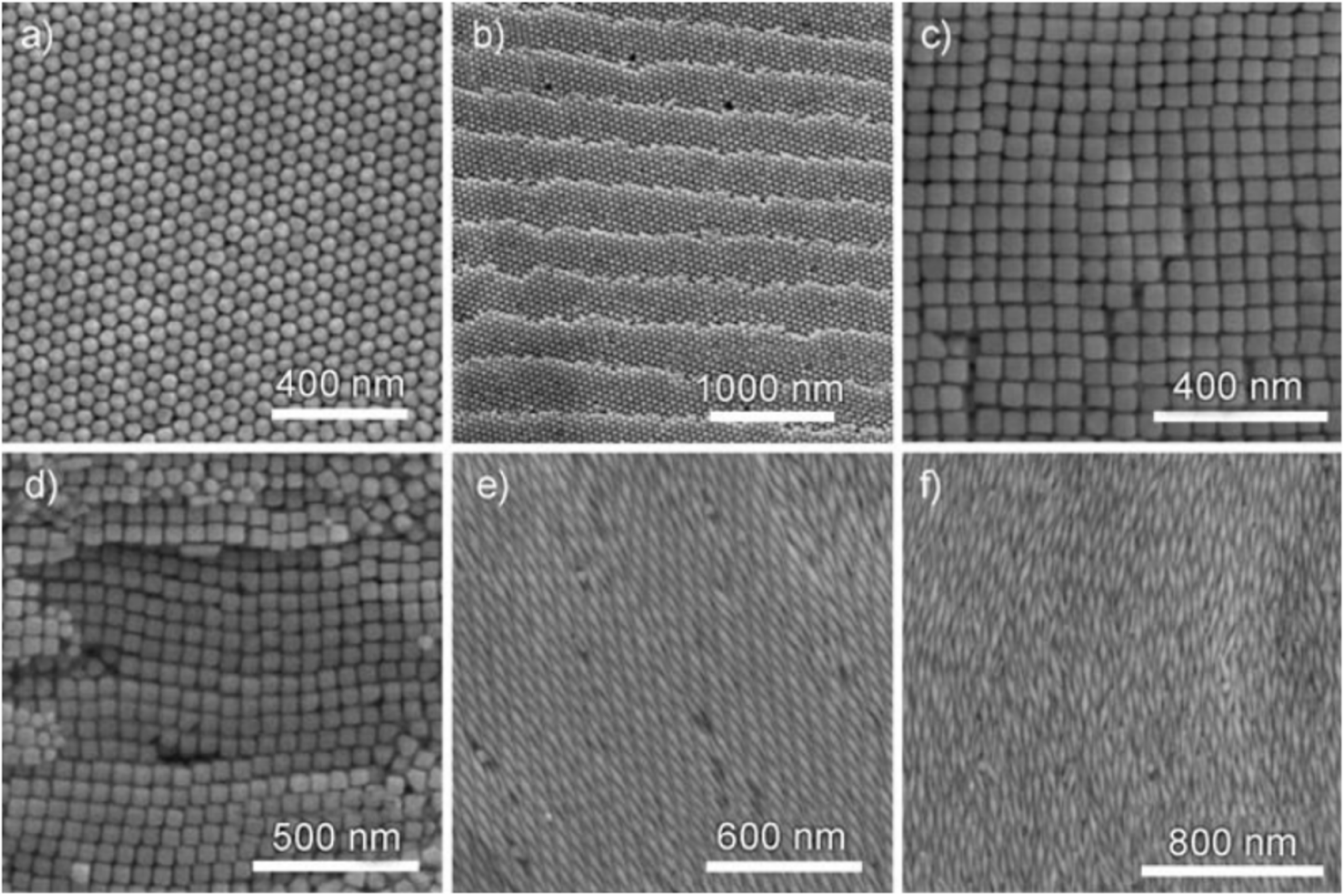
SEM images of Au nanoassemblies. (a) Hexagonally filled Au polyhedra. (b) Hexagonally filled Au polyhedra with periodic monosteps. (c) Tetragonally packed Au nanocubes. (d) Nanocubes stacked in layers. (e) A 3D ordered superstructure formed of Au bipyramids. (f) Nematic structure formed of Au bipyramids. Figure 17a–f was reproduced from [107], T. Ming. et al., “Ordered Gold Nanostructure Assemblies Formed By Droplet Evaporation”, Angew. Chem., Int. Ed., with permission from John Wiley and Sons. Copyright © 2008 Wiley-VCH Verlag GmbH & Co. KGaA, Weinheim. This content is not subject to CC BY 4.0.

Reports on the relative radiative and non-radiative damping contributions to plasmon decay in nanorods and nanospheres revealed that the magnitude of the scattering cross section (and hence PT conversion) depends on size and shape rather than the volume, with smaller particles being efficient absorbers and larger nanoparticles effective scatterers [92]. The comparison between Ag, Pt, and Pd confirmed this. Non-radiative decay was absent in Ag above a certain particle size, while Pt and Pd exhibited non-radiative decay for all sizes, confirmed through conductivity measurements on plasmonically generated hot electrons. Another comparison between plasmonic heat generation in Au nanorods and spheres revealed a much higher (60%) heating efficiency for nanorods, due to the presence of more atoms near the surface (due to the flattened morphology of rods compared to spheres, Figure 17) which contribute more to the heating effect than the interior atoms that do not participate in the resonance [107]. Such improvements to PT heating because of a higher surface-to-volume ratio has also been studied with shapes such as rings, ellipsoids, rods, and disks [96], indicating another possible approach to maximize PT efficiency.

Remarkable changes to absorbance manifest also through changing the proximity between nanoparticles. Since the concentrated electric field distributions during SPR extend well outside the physical boundaries of the plasmonic structures, their influence on adjacent particles can lead to interesting resonance phenomena. As an example of such interactions, the most common arrangement of a nanoparticle dimer is often considered. In general, the interactions are classified into repulsive and attractive, wherein the former blueshifts the resonance with an associated increase in damping. In contrast, attractive repulsions lead to an increase in resonance intensity coupled with a redshift. Multiple phenomena come into play while considering the coupling between nanoparticles, such as separation distances, morphologies, and the influences of dipole–dipole/dipole–multipole/multipole–multipole coupling, to name a few.

Generating a universal model that describes plasmon coupling is not possible. As an example of this, the conventionally used dipole approximation, which predicts the decay of the near field fails for small interparticle distances (at separation-to-diameter ratios less than 0.1). For such cases, alternate models explain the observed results, such as the plasmon hybridization model and extended multipole methods. The plasmon hybridization model, for example, considers the interactions at such distances through the formation of bonding and anti-bonding plasmon modes, yielding a robust prediction of the resonance shifts [108–109]. As the particles approach, more modes occur, indicating that the system's symmetry reduces to a side-to-side approximation. When two nanoparticles interact, their unique modes pair to form new dipole resonances of different energies. For example, in nanorods arranged end-to-end, the net dipole moment of the higher energy mode is zero. In an asymmetric nanorod dimer (heterodimer), both bonding and anti-bonding plasmon modes occur at the same time, resulting in a non-zero dipole. The fractional shift in resonance wavelength depends on the size (*S*), the separation between the nanoparticles (*R*), and shape factors (Λ, γ):


[43] $$\frac{\Delta \lambda }{\lambda }=\frac{1}{12\Lambda {\left(1+R/S\right)}^{3}-\left(1+\gamma \right)}.$$

**Figure 18:**
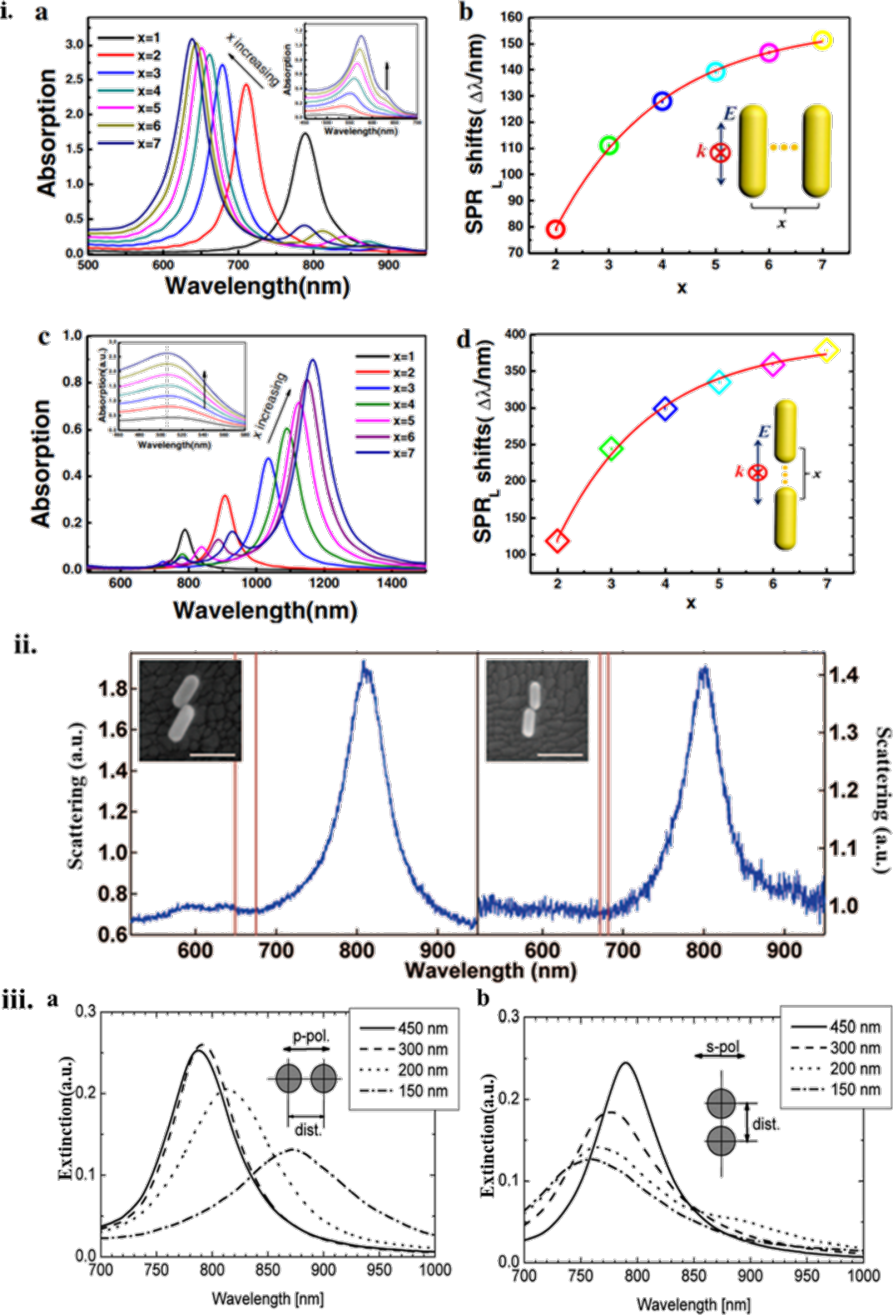
(i) FDTD calculation of absorption spectra of Au nanorods (aspect ratio of ca. 3) for different numbers (*x*) of nanorod assemblies: (a) Side-side assembly. (b) SPR peak shift for side-by-side assembled Au nanorods. (c) End-to-end assembly. (d) SPR peak shift for end-to-end assembled Au nanorods. Figure 18 panel i was reprinted by permission from Springer Nature from [110] (“Plasmonic Properties of the End-to-End and Side-by-Side Assembled Au Nanorods” by J. Liu et al., Plasmonics, Vol. 10, pp. 117–124, 2015), Copyright 2015 Springer Nature. This content is not subject to CC BY 4.0. (ii) Scattering spectra of Au rods in different orientations (aspect ratio of 2–2.7) on ITO substrates with interparticle distances smaller than 1 nm. Figure 18 panel ii was reprinted with permission from [111], Copyright 2009 American Chemical Society. This content is not subject to CC BY 4.0. (iii) Extinction spectra of a 2D array of the Au nanoparticle pairs with interparticle distances from 450 to 150 nm: (a) Parallel to the long particle pair axis and (b) normal to this axis. Figure 18 panel iii was reprinted from [109], Optics Communications, vol. 220, by W. Rechberger; A. Hohenau; A. Leitner; J. R. Krenn; B. Lamprecht; F. R. Aussenegg, “Optical properties of two interacting gold nanoparticles”, pages 137–141, Copyright (2003), with permission from Elsevier. This content is not subject to CC BY 4.0.

A comprehensive treatise of the fascinating topic of plasmon coupling is outside the scope of this review, and hence only a rough overview of the differences in resonance due to particle coupling is presented in Figure 18.

**Figure 19:**
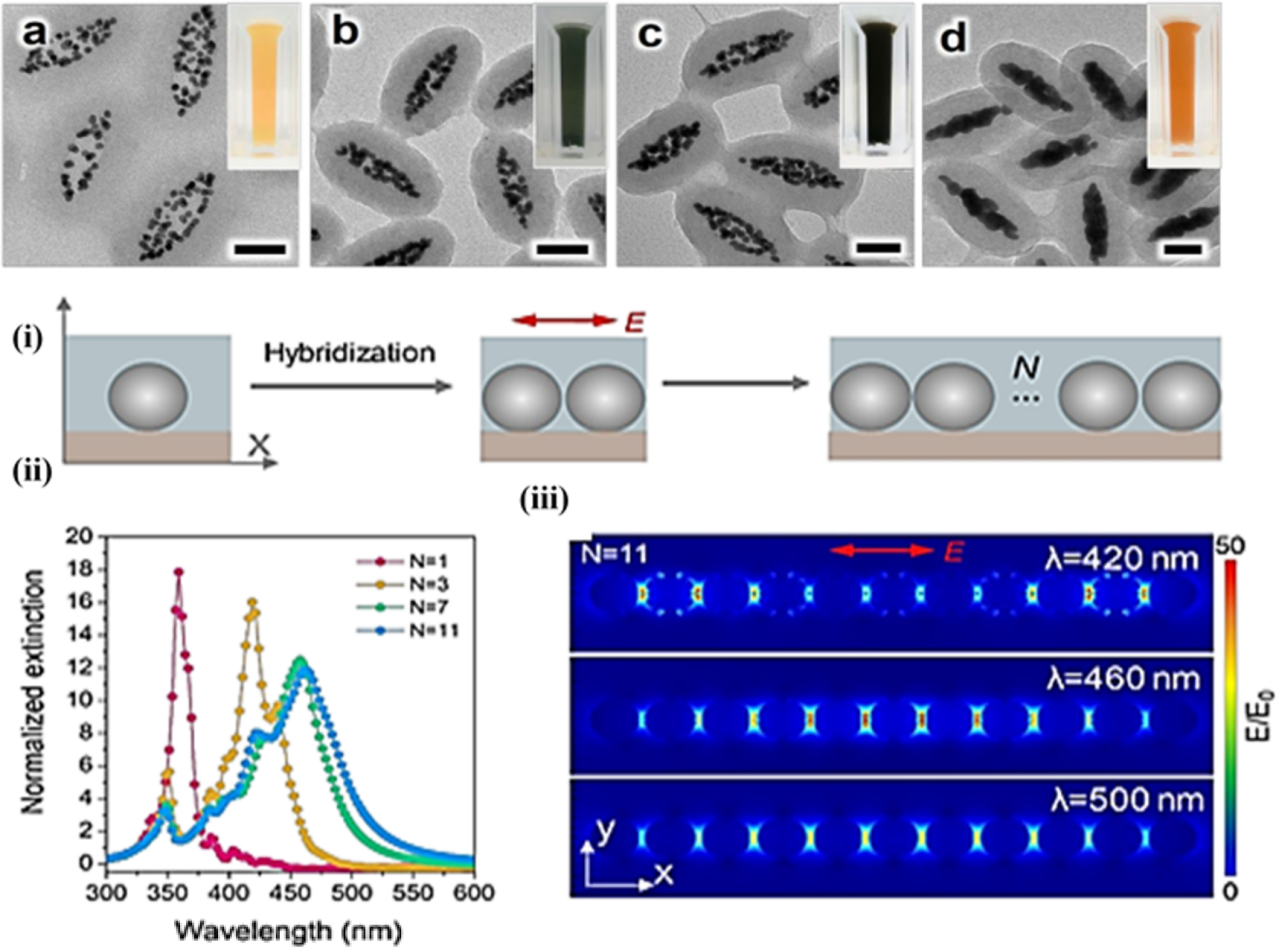
TEM images with 50 nm scale bars of linked Ag nanospheres prepared with an injection rate of 5 μL/min and for different concentrations of the precursor solution: (a) 15 μL, (b) 25 μL, (c) 35 μL, and (d) 65 μL. (i) Schematic diagram showing the model considered in finite-difference time-domain (FDTD) simulations. (ii) Simulated and normalised extinction graph for Ag nanoparticles for different chain lengths. (iii) Simulated FDTD results for the enhancement for the electric near field along the Ag nanoparticle chain (*N* = 11) excited by incident light of different wavelengths (420, 460, and 500 nm). Figure 19 panels i–iii were adapted with permission from [114], Copyright 2019 American Chemical Society. This content is not subject to CC BY 4.0.

Excellent research that has treated this topic can be found in [108,111–112] and is partially shown in Figure 18. It is evident that a significant tuning of the absorption range of plasmonically coupled nanostructures is possible and highly beneficial. Another important consideration are the changes from non-radiative to radiative damping that occur for coupled and uncoupled nanostructures, respectively. Repulsive coupling, in particular, can be postulated to lead to a higher extent of non-radiative decay and a higher heating efficiency. Considerable differences to heating will arise when the nanostructures are supported on solid supports, such as membranes, compared to when they are dispersed in a liquid phase. Plasmonically excited and coupled nanoparticles are vital to achieving controllable and efficient conversion into heat. Specifically, interparticle coupling of the field intensities can shift the far-field and the near-field scattering (the latter becoming important for interparticle distances less than 10 nm for Au nanorods, for example) of the plasmonic absorption considerably [113]. A sophisticated seeded technique of nanoparticle synthesis led to the creation of linked Ag nanospheres with differing numbers of proximal nanoparticles that formed a chain. A clear enhancement in the absorbance to the entire UV–visible range was observed for linked particles as compared to separated particles. A steam generation efficiency of 95% was observed for the best samples, with increases in water temperature of up to 100 °C on irradiation with a Xenon lamp as shown in Figure 19.

When comparing Ag and Au with the less commonly used Pt, Pt has been proven to be more effective than gold nanoshells (AuNSs), a commonly used morphology for PT heating. The loss of the specific morphology of the AuNSs, whereas Pt nanospheres maintained their shape, indicates a better suitability of Pt nanospheres for applications requiring morphological stability at temperatures up to 700 °C [115].

Palladium has a tunable plasmon response in the UV–visible–NIR region with high PT conversion efficiency and PT stability [116], the latter of which originates from its high melting point and the correspondingly higher capacity to withstand high irradiation fluences compared to Ag and Au nanoparticles [117]. This has been confirmed in a study wherein Pd nano hexagonal sheets preserved their morphology unlike Au and Ag nanoparticles (spheres) when exposed to the same laser fluence. Another significant outcome of this work was the substantial tuning of the LSPR absorption (by more than 250 nm) by changing the edge lengths by a few tens of nanometres [118]. Capping agents, growth directing agents, and surfactants were optimized to achieve homogeneity, size controllability, and absorbance tuning. Although the absorption of these nanostructures were in the NIR region, a tuning towards the UV–visible region was possible through the change in thickness of the sheets [119]. An increase in temperature by 20 °C of an aqueous solution with the dispersed nanosheets and irradiated with a laser confirmed their relevance for PT applications.

The plasmonic properties of copper have been investigated extensively through theoretical approaches, but practical uses for plasmonic applications have been overshadowed by its counterparts (Ag and Au). Copper has been shown to be quite attractive for PT energy conversion, wherein differences in morphology control the light trapping efficiency. Facile one-step laser ablated copper can form hierarchical nanostructures with controlled morphology, depending on the laser scanning rate. Absorbance over a broad range between 200 and 800 nm and with efficiencies over 60% has been reported [21,90].

Similar to the coinage metals copper, silver, and gold, aluminium is a highly promising material for exciting strong surface plasmon resonances in the visible and ultraviolet range, as shown in Figure 15. It is used in plasmonic devices [120] such as resonators [121–122], antennas [123], and biosensors [124]. Aluminium absorbs photons in a wavelength range outside that of conventional plasmonic nanomaterials, which can be exploited for PT applications, such as for absorbing this inaccessible part of the solar spectrum. Al also has a very small scattering cross section, resulting in a much higher PT conversion effect compared to, for example, Au and Ag. Even though Al is plasmonic, the absorption cross section is too low. This shortcoming can be circumvented by using morphologies other than spheres, which can minimize scattering, and through increasing the concentration of the particles [125].

Magnesium nanoparticles have multiple resonances across the UV–vis–IR range [126]. One possible shortcoming for Mg is that they are less lossy than Al and Ga [127], which limits their potential for PT heating. Another shortcoming is the easy oxidation of Mg. Although it does not detrimentally affect the plasmonic properties it reduces considerably the thermal conductivity, leading to a reduced PT performance. The potential of Mg NPs for PT heating, hence, remains unexplored. Of all plasmonic metals considered so far, Na and K have been outliers regarding a study for PT applications [60]. Plasmonically, the very high quality factors of Na and K limit their use for these applications as they are less lossy. Practically, the extremely reactive nature of both metals severly restricts their use.

Tellurium nanoparticles, when engineered appropriately, can have broadband UV–vis plasmonic absorption resonances and are also stable against oxidation, presenting good potential for long term PT applications [35]. They have been proven to lead to higher temperature increases than even perfect absorbers such as TiO*_x_*, with an 85% absorbance reported in the region between 300 and 2000 nm. A fundamental analysis of the dielectric function revealed a combination of plasmonic (UV region) and Mie-type multipolar resonances (for particles of sizes from 0.1λ to λ) to be the reason for the broadband optical absorption. The wide size distribution (10 to 300 nm) of laser-ablated Te nanoparticles allowed for broadband absorption, along with the relatively small bandgap (0.35 eV), which also aids in longer wavelength absorption. Such an optical duality, wherein plasmonic absorption (typically revealed as the most efficient absorption of light) and other resonance modes can be coupled, can prove to be a very effective means of realising the ideal nanomaterial for PT heating [128].

Molybdenum exhibits tunable LSPR between 600 and 1200 nm (for a nanosheet morphology) and has potential in PT studies such as in PT therapy. The lower melting point of Mo, compared to Pd, Au or Ag, is a possible shortcoming regarding PT applications, as laser fluences often lead to thermalization temperatures approaching 1000 K [129]. A comprehensive compilation of the different morphologies of metallic nanostructures explored in PT conversion and the associated data, such as particle dimensions, maximum temperatures/temperature increases achieved, as well as the irradiation means is presented in Table 1. The temperatures in the individual studies have been measured using equipment such as thermal imagers [128–129], thermocouples [130], and IR cameras [131]. Predictive calculations of the final temperatures through calculations [132–133] based on the radiation intensity and the material properties (dielectric constant) have also been performed.

**Table 1:** The PT conversion performance of metal nanostructures of different morphologies, such as nanosheets, nanostars, nanospheres, core–shell NPs, nanorod, nanocubes, nanoflowers, nanocages, and thin films, using different laser power intensities, side lengths (*a*), radii (*R*), aspect ratios (*A*), and thicknesses (*T*).

No.	Material	Morphology	Dimensions	Rise in temperature (K)	Irradiation/power	References

1	Au	core–shell	—	115–135	solar radiation	[130]
2	Au	spheres	*R* = 100 nm	80	laser/1 mW	[96]
3	Au	spheres	*R* = 20 nm	27.7	laser /0.12 W	[134]
4	Cu	nanoflowers	—	49	laser/1 kW	[90]
5	Te	spheres	*R* = 150 nm	85	tungsten lamp/78.9 W	[128]
6	Ag	nanocubes	*a* = 75 nm	227	laser/300–800 mW	[135]
7	Au	nanorod	*A* = 4.5 nm	55	laser/1 W	[136]
8	Pd	nanosheets	*L* = 41 nm	48.7	laser/1 W	[119]
9	Ag	assemblages	domains = 30 nm	105	xenon lamp/300 W	[114]
10	Pt	spheres	*R* = 50 nm	300	laser/375 mW	[115]
11	Ge	sphere	*R* = 80 nm	14	simulated light/80 mW	[137]
12	Ge	sphere	*R* = 30 nm	121	xenon lamp/8.75 mW	[138]
13	Au	spheres	*R* = 10 nm	85	laser/30 mW	[105]
14	Au	spheres	*R* = 25 nm	75	laser/1 mW	[105]
15	Au	spheres	*R* = 5 nm	15	laser/20 mW	[96]
16	Au	spheres	*R* = 15 nm	13	laser/1000 W	[139]
17	Au	nanorod	*A* = 4 nm	55	laser/1 sun illumination	[101]
18	Au	spheres	*R* = 40 nm	17–26	laser/1–10 kW	[140]
19	Mo	spheres	*R* = 20 nm	41	—	[141]
20	Au	nanoarray	*R* = 20 nm	—	laser/7.3–15.7 kW	[141]
21	Au	spheres	*R* = 50 nm	52	laser/20.5 µW	[142]
22	Ag	nanoshell	*R* = 25 nm	—	laser/1 mW	[143]
23	Au	spheres	*R* = 40 nm	60	laser/400 mW	[81]
24	Au	bipyramid	—	95	—	[144]
25	Au	thinfilm	*T* = 120 nm	94	laser/3.5 W	[145]
26	Au	nanostoves	*R* = 5 nm	65	laser/3.8 kW	[146]
27	Au	nanocylinder	*L* = 10 nm	20	laser/0.1 mW	[147]
28	Au	spheres	*R* = 40 nm	33	laser/3 mW	[148]
29	Au	nanorods	*A* = 7 nm	45	laser/1 mW	[149]
30	Au	spheres	*R* = 20 nm	0.7	laser/9 µW	[150]
31	Au	spheres	*R* = 10 nm	25	laser/5 mW	[151]
32	Au	spheres	*R* = 30 nm	4	laser/10 kW	[8]
33	Au	core–shell	*R* = 110 nm	10	laser/0.4 W	[88]
34	Au	nanocages	inner edge length = 30 nm, thickness = 5 nm	—	laser/1.5 W	[152]
35	Pd	nanosheets	*T* = 80 nm	53	laser/0.3 W	[153]
36	Pd	porous nanoparticles	Pd of size = 58 nm, porous size of 8 nm	50	laser/0.5 W	[154]
37	Pd	nanosheets	*T* = 5 nm	50	laser/0.14 W	[155]
38	Pd	nanosheets	*T* < 8 nm	51	laser/0.5–1 W	[156]
39	Pd	nanosheets	*T* = 100 nm	49.8	laser/1 W	[157]
40	Pd	porous nanoparticles	*R* = 96–153 nm	53	laser/4 W	[158–159]
41	Au	spheres	*R* = 18 nm	57.8	laser/5.09 kW	[131]
42	Au	spheres	*R* = 3–40 nm	24–51.9	laser/10 kW	[140]
43	Au	spheres	*R* = 8.5–138.9 nm	5–15	laser/1.5 sun illumination	[160]
44	Ag	membrane	*T* = 60 nm with a porous size of 0.40 nm	42	laser/23 kW	[132]
45	Ag	membrane	—	2.5	laser/660 W	[161]
46	Pd	nanocomposites	size = 31 nm	51	laser/1.5 W	[119]
47	Pd	nanoflowers	size = 22 nm	62	laser/4 W	[118]
48	Au-PEG-Ce6	nanostars	size = 54 nm	51	laser/1 W	[162]
49	Pd@Pt-PEG-Ce6	nanocomposites	*T* = 80 nm	40	laser/0.5 W	[163]
50	Pd@COS-RGD	membrane	size = 23 nm	60.9	laser/3 W	[164]
51	CD-Pd	nanosheets	*L* = 50 nm, *t* = 14 nm	23	laser/1 W	[164]
52	Pd@Ce6	spheres	*R* = 116 nm	35	laser/4 W	[165]
53	Pd -PEI-Ce6	nanosheets	*T* = 2.2 nm	4	laser/0.5 W	[166]
54	PdC-HSA-ICG	spheres	*R* = 55 nm	50.4	laser/0.5 W	[167]
55	Pd-cys@MTX-RGD	nanosheets	*T* = 24 nm	50	laser/1 W	[168]
56	Au-DNA	nanostoves	*R* = 5 nm	53	laser/3.8 kW	[147]
57	Au-PCR	thinfilm	*T* = 10–120 nm	94	laser/3.5 W	[146]

Understanding the features of different morphologies, how the materials interact with other substances, and eventually how they might be used in PT applications requires primarily a knowledge of the dimensions of nanoparticles and their effects on SPR and PT performance. Depend on the PT applications, particle morphologies have to be chosen to obtain desired attributes such as absorption range, modality of dispersion, cyclability, and required temperature while also keeping in mind the practical aspects such as cost and ease and repeatability of synthesis. In Tables 1–5 (above and below), we have summarized the most commonly investigated morphologies for various classes of materials.

#### 4.2 Multielemental nanomaterials

**Figure 20:**
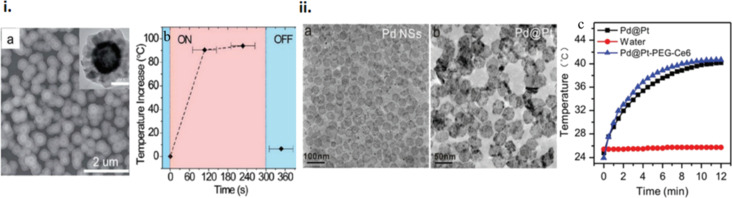
TEM images of CuS, metal, and bimetal plasmonic nanoparticles and the corresponding temperature rise. (i) (a) SEM and TEM image of hierarchical Cu_7_S_4_@ZIF8 core–shell nanostructures. (b) Temperature rise of Cu_7_S_4_@ZIF8 nanoparticles under a laser power irradiation of 100 mW/cm^2^. Figure 20 panel i a,b was adapted from [176] (“Photothermal-enhanced catalysis in core-shell plasmonic hierarchical Cu_7_S_4_ microsphere@zeolitic imidazole framework-8”, © 2016 F. Wang et al., published by The Royal Society of Chemistry, distributed under the terms of the Creative Commons Attribution 3.0 Unported License, https://creativecommons.org/licenses/by/3.0/). (ii) (a) TEM image of Pd nanosheets. (b) TEM image of Pd@Pt nanoplates. (c) Temperature rise of Pd and Pd@Pt nanostructures under a laser intensity of 0.5 mW/cm^2^. Figure 20 panel ii a–c was adapted from [163], J. Wei et al., “A Novel theranostic Nanoplatform based on Pd@Pt-PEG-Ce6 for Enhanced Photodynamic therapy by modulating tumor Hypoxia microenvironment”, Adv. Funct. Mater., with permission from John Wiley and Sons. Copyright © 2018 WILEY-VCH Verlag GmbH & Co. KGaA, Weinheim. This content is not subject to CC BY 4.0.

The manipulation of morphology leading to significant enhancements in PT properties has been researched through various modalities. One such approach is the fabrication of hollow micro/meso- and nanoporous materials, which have benefits in terms of a lower density (negating sedimentation issues when used for water vaporisation for example) than their solid counterparts, enhanced light absorptivity through internal reflection and increased electron scattering, and the important advantage of having a much higher area of vaporisation (as the pore surfaces also act as vaporisation centres) [169]. Novel nanomaterials, such as mesoporous Au/Ag nanostructures, have been investigated. The bimodal pore size distribution (11 and 3 nm), in addition to enhancing the formation of vapour in the nanoparticle interior and allowing the vapour to diffuse faster to the surface, enhance electron scattering (and hence electron–phonon coupling) by acting as a dense collection of scattering centres, reducing considerably the mean free path of electrons. This has led to a fivefold increase in PT heating (calculated from the ratio of the vapour generation rate to the input energy) compared to a solid Ag/Au core–shell morphology. For example, a Pd@Pt bimetal material for vapour generation is shown in Figure 20 and below in Figure 22. A compilation of the different morphologies and temperature increases for metal–metal and metal–semiconductor nanostructures is presented in Table 2.

**Table 2:** The PT conversion properties of nanostructures of metal–metal as well as metal–semiconductors. A denotes edge length, *R* is the particle radius, *A* is the aspect ratio and *T* the thickness.

No.	Material	Morphology	Dimensions	Rise in temperature (K)	Irradiation/power	References

1	Pd@Ag	nanoplates	*T* = 5.4 nm	50.8	laser/1.4 W	[170]
2	Ag@Si	thinfilm	*T* = 150 nm	230	laser/2 W	[85]
3	Pd@Si	nanosheets	*T* = 32 nm	48.4	laser/1.4 W	[171]
4	Porous Pd@Pd	nanocubes	*a* = 22.8 nm	36	laser/8 W	[102]
5	Pd@Au	nanoplates	*T* = 15 nm	51	laser/0.3 W	[172]
6	Pd@Au	sandwich like structure	size = 253.7 nm	55	laser/2.4 W	[173]
7	Au-Ag	nanofluids	*R* = 10 nm for Au *R* = 30 nm for Ag	62	laser/10 sun illumination	[174]
8	Au@Pt	nanorods	*A* = 3.5 nm	61.4	laser/2.4 W	[175]

The mechanism of self-doping, that is, the manipulation of charge carrier density through creation of vacancies is a crucial means of realizing plasmonic tuning, not possible with nanomaterials composed of a single element [177]. An important example of a self-doped plasmonic tunable material is copper sulfide, and its PT conversion is shown in Figure 20. Copper sulfide in its pristine form does not exhibit strong plasmonic absorbance due to the overlap with interband transitions [178]. However, removing Cu atoms through various methods, such as annealing, results in a multitude of non-stoichiometric stable chemistries, such as covellite (CuS), anilite (Cu_1.75_S), digenite (Cu_1.8_S), djuleite (Cu_1.96_S), and chalcocite [179] (Cu_2_S), all of which have LSPRs in the NIR due to decreasing amounts of negatively charged copper vacancies (with an associated decrease in electron densities) [180]. PT applications have benefitted from plasmonic Cu_2_S, an example of which are CuS nanoflowers supported on a membrane for solar vapour generation. High PT conversion efficiencies (up to 81.2%) were reported, due to the joint contributions from a broadband absorption as well as dispersion on a membrane on the surface (as compared to a vapour generation system based on a CuS nanofluid). Further efforts on PT heating with Cu_2_S-based materials are highlighted in Table 3.

**Table 3:** The PT conversion properties for copper sulfide nanostructures of different morphologies like spheres and nanodots using different laser power intensities, where *R* is the particle radius.

No.	Material	Morphology	Dimensions	Rise in temperature (K)	Irradiation/power	References

1	CuS	spheres	*R* = 20 nm	12.7	laser/40 W	[181]
2	Cu_2−_*_x_*S	nanodots	*R* < 5 nm	19	laser/1.41 W	[182]
3	Cu_7_S_4_	spheres	*R* = 5.9 nm	—	—	[183]

**Figure 21:**
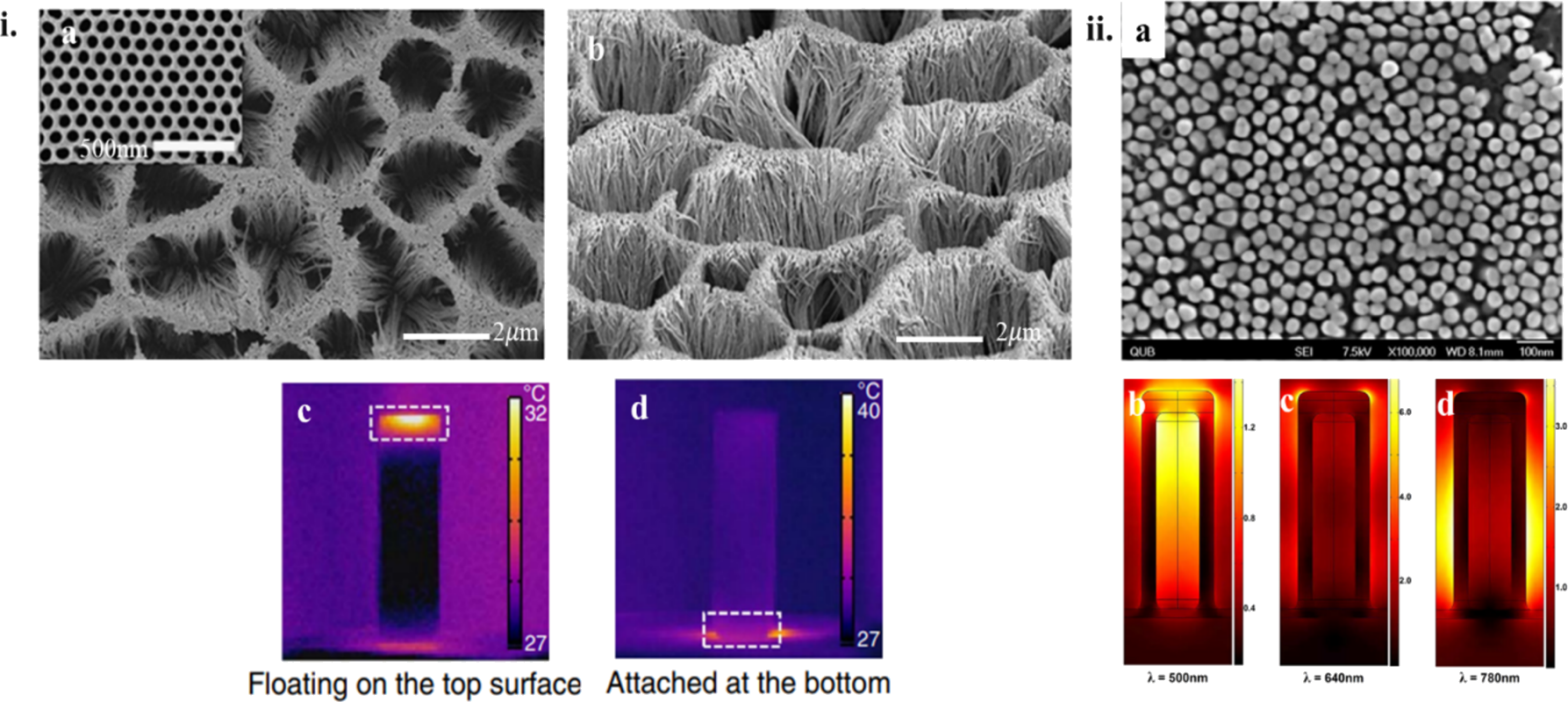
(i) (a, b) SEM images of a black Au membrane in a hexagonal ordered array of AAO. (c) Thermal images of the black gold membrane floating on water in a cuvette and (d) at the bottom of the cuvette under an irradiation density of 10 kW/m^2^. Figure 21 panel i a–d was adapted from [184] (© 2015 K. Bae et al., distributed under the terms of the Creative Commons Attribution 4.0 International License, https://creativecommons.org/licenses/by/4.0). ii. a. SEM image of AAO capped Au nanotube array. b.–d. Simulated electric field intensity of AAO capped Au nanotube array at different wavelengths (the nanotubes are 120 nm long with a 24 nm inner diameter, 8 nm wall thickness and 60 nm spacing. Caps are 12 nm thick. Figure 21 panel ii a–d was adapted from [185] (W. R. Hendren et al., “Fabrication and optical properties of gold nanotube arrays”, J. Phys.: Condens. Matter, vol. 20, article no. 362203, published on 14 August 2008; DOI: 10.1088/0953-8984/20/36/362203); © 2008 IOP Publishing. Reproduced with permission via Copyright Clearance Center. All rights reserved. This content is not subject to CC BY 4.0.

In a similar multielemental PPT conversion study, pore-widened anodic aluminum oxide (AAO) templates as shown in Figure 21 were used to synthesize capillary force-driven self-agglomerated Al_2_O_3_/Au membrane nanomaterials composed of nanowires of this composite. The resulting structures exhibited a multitude of PT heating enhancing factors, namely nanoscale gaps of different sizes, which enable broadband absorption (91% absorption between 400 and 2500 nm due to gaps varying from zero to hundreds of nanometers over a few micrometres), low taper angles (ca. 1°) for sustenance of the plasmon wave throughout the nanowire, thereby avoiding reflection losses, low reflection of incident light (7% between 2.5 and 17 µm), and hydrophilicity, which ensures continuous contact of water during the evaporation process. All of these factors contributed to a high solar thermal conversion efficiency of 57% with an irradiance of 20 kW/m^2^.

#### 4.3 Doped metal-oxide plasmonic nanomaterials

Metal oxide nanostructures are suitable for PT applications because of physiochemical stability and increased availability, in addition to prospects of self-doping through the creation of oxygen vacancies. Specifically, certain oxygen-deficient non-stoichiometric metal oxides [186] such as WO_3−_*_x_*, MoO_3−_*_x_* and TiO*_x_* with a strong absorption across the UV–NIR range with good thermal stability are being researched on increasingly. For example, titanium oxide, with a bandgap energy of 3 eV in the far UV, can be tailored for absorption in the visible region by doping with other transition metals with high efficacy for PT applications [34,187–189]. However, heavily doped or self-doped TiO_2_ in itself is plasmonic, as the self-doping process (due to vacancy creation) leads to excess free electrons due to oxygen desorption. It has been calculated that if free carrier densities exceed 10^20^ cm^−3^, materials can become plasmonic in the NIR region (the relation between the material and medium dielectric constants notwithstanding). Confirming this postulation, TiO_2_ exhibits plasmonic absorption when the ratio between oxygen and Ti atoms reaches approximately 1.67:1. Also, through a broad size distribution of the particles, optical absorption efficiencies up to 90% across the solar spectrum can be reached. The added advantage of TiO_2_ being a high-loss material makes it a great candidate for PT heating applications, evidenced by a temperature increase of 50 °C under sunlight of the aqueous solution in which they were dispersed [190].

**Figure 22:**
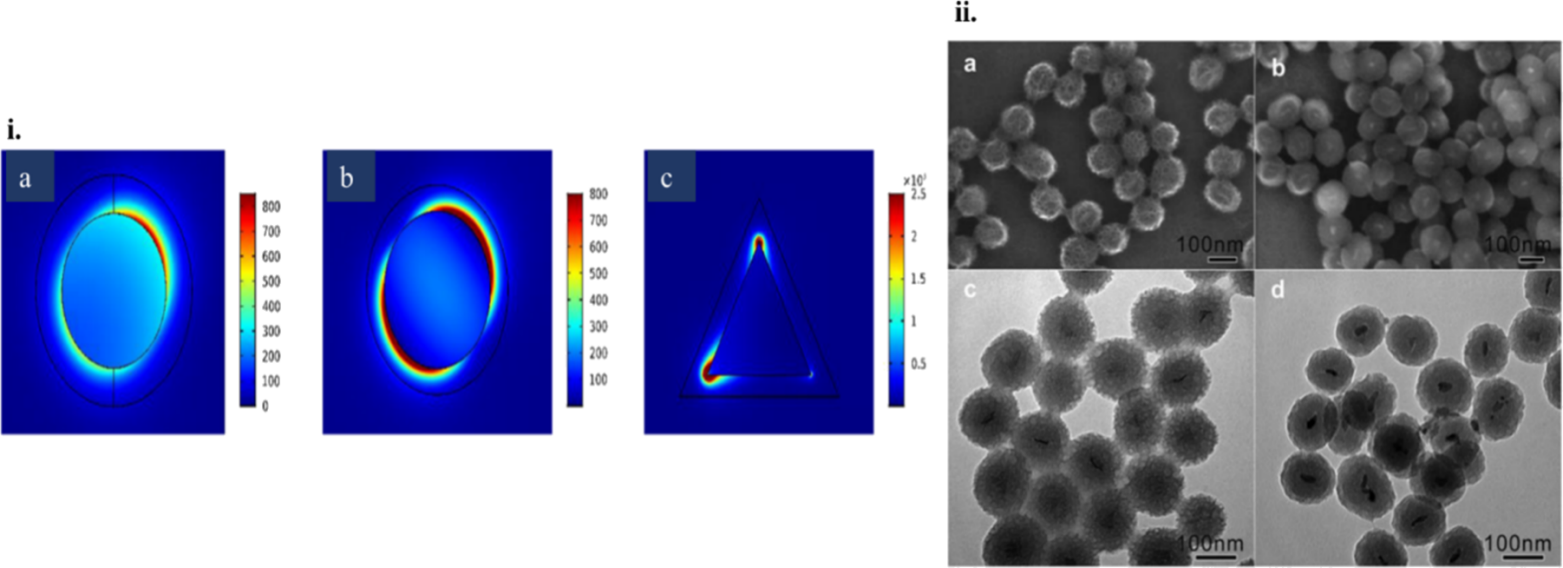
(i) Simulated electric field intensities of Ag/SiO_2_ at the ZX plane: (a) Core–shell (*d*_Ag_ = 40 nm, *t*_SiO2_ = 10 nm). (b) Nanodisk (*d*_Ag_ = 60 nm, *t*_Ag_ = 5 nm, and *t*_SiO2_ = 10 nm). (c) Nanoprism (*l*_Ag_ = 70 nm, *t*_Ag_ = 5 nm, and *t*_SiO2_ = 10 nm. Figure 22 panel i a–c was reproduced from [133], A. R. Mallah et al., “An innovative, high-efficiency silver/silica nanocomposites for direct absorption concentrating solar thermal power”, Int. J. Energy Res., with permission from John Wiley and Sons. Copyright © 2020 John Wiley & Sons, Ltd. This content is not subject to CC BY 4.0. (ii) TEM images of (a, c) Pd@Ag@mSiO_2_ and (b, d) Pd@Ag@mSiO_2_-Ce_6_. Figure 22 panel ii a–d was reproduced with permission of The Royal Society of Chemistry, from [191] (“Photothermally enhanced photodynamic therapy based on mesoporous Pd@Ag@mSiO_2_ nanocarriers”, by S. Shi et al., J. Mater. Chem. B, vol. 1, issue 8, © 2013); permission conveyed through Copyright Clearance Center, Inc. This content is not subject to CC BY 4.0.

Another interesting photocatalytic PPT study with an additional layer of SiO_2_ is shown in Figure 22. This layer prevented charge hot carrier injection from TiO_2_, ensuring energy dissipation of these carriers as heat and also protecting the AgNPs from oxidation and chemical poisoning. The temperature increase provided by the plasmonic heating improved the hydrogen generation rate from water by fivefold. Since hydrogen generation is a common application in the investigation of the photocatalytic properties of TiO_2_, such combinatorial effects can have benefits such as improving hydrogen yield, minimizing the footprint and cost associated with energy production and desalination, as well as improving the robustness of the materials.

Combining metals with metal oxides can lead to multiple benefits, which has led to extensive studies regarding catalysis, sensing, energy storage, water splitting, and solar cells, to name a few. By extension, the enhanced properties of such composites such as enhanced absorption, broadband absorption, enhanced material stability, and cycling performance have direct relevance for PT applications. One study has investigated the combinatorial effects of AuNPs [192] as a PPT converter, TiO_2_ as a photocatalyst as well as an internal light scattering enhancer for desalination, and metal oxide nanostructures for PT applications. The enhancements to the photocatalytic function as well as solar thermal conversion makes such composites attractive for PT applications. A comprehensive summary of different metal–metal oxide composites that have been investigated for PT conversion are presented in the Table 4.

**Table 4:** PT conversion properties of different morphologies and compositions of metal–metal oxide nanostructures for different irradiation intensities. *R* is the particle radius, and *T* is the thickness.

No.	Material	Morphology	Dimensions	Rise in temperature (K)	Irradiation/power	References

1	WO_3_	nanosheets	size = 150 nm, *T* = 5 nm	61	laser/10 kW	[193]
2	Ti_2_O_3_	spheres	*R* = 300 nm	70	laser/7 kW	[194]
3	TiO_2_	nanorod	*A* = 3 nm	200	laser/400 mW	[66]
4	Au-Silica gel	Au nanoflowers in silica matrix	Au of *R* = 30 nm, porous size of silica is 3.5–4 nm	50	laser/1 kW	[195]
5	Au@GO	Au-Nanorod @GO nanosheet	Au of *A* = 3.3 nm	53.5	laser/1 kW	[196]
6	Ag@Si	Thinfilm	*T* = 150 nm	230	laser/2 W	[85]
7	Au@ TiO_2_	core–shell	Au of *R* = 30 nm, TiO_2_ of *R* = 50 nm	—	laser/5 kW	[192]
8	Au/TiN	nanofluids	Au of *R* = 3–7 nm, TiN of *R* = 20 nm	40	laser/720 W	[197]
9	Au@ Al_2_O_3_	Au nanoislands in Al_2_O_3_	*R* = 20 nm, *T* = 30 nm	90	laser/11 W	[198]
10	Au-PEG-Ce6	nanostars	size = 54 nm	51	laser/1 W	[162]
11	Au@ SiO_2_	core–shell	*R* = 62 nm, *R* = 77 nm	2	—	[93]
12	Au@ SiO_2_	core–shell	*R* = 40 nm, *R* = 40 nm	—	—	[199]
13	Ag@ TiO_2_	core–shell	10–20 µm	52	laser/1.5 sun illumination	[200]
14	Al-Ti-O	membrane	*T* = 5 nm	—	laser/820 W	[201]
15	Ag@ TiO_2_	core–shell	*R* = 19 nm of Au, porous size of 220 nm of TiO_2_	80	laser/1 kW	[202]
16	Ag@ silica	core–shell	Ag disk *R* = 30 nm, *t* = 10 nm @ SiO_2_ with *T* = 10 nm	30.15	laser/50 sun illumination	[133]
17	Au@ SiO_2_	nanoflowers	30 nm nanoflowers at 3 nm porous of SiO_2_	40	laser/1 kW	[195]
18	Au@ AAO	matrix	Au of *R* = 30 nm	80	laser/20 kW	[184]
19	Pd@ SiO_2_@ Ag	membrane	size = 164 nm	6	laser/1 W	[191]

**Figure 23:**
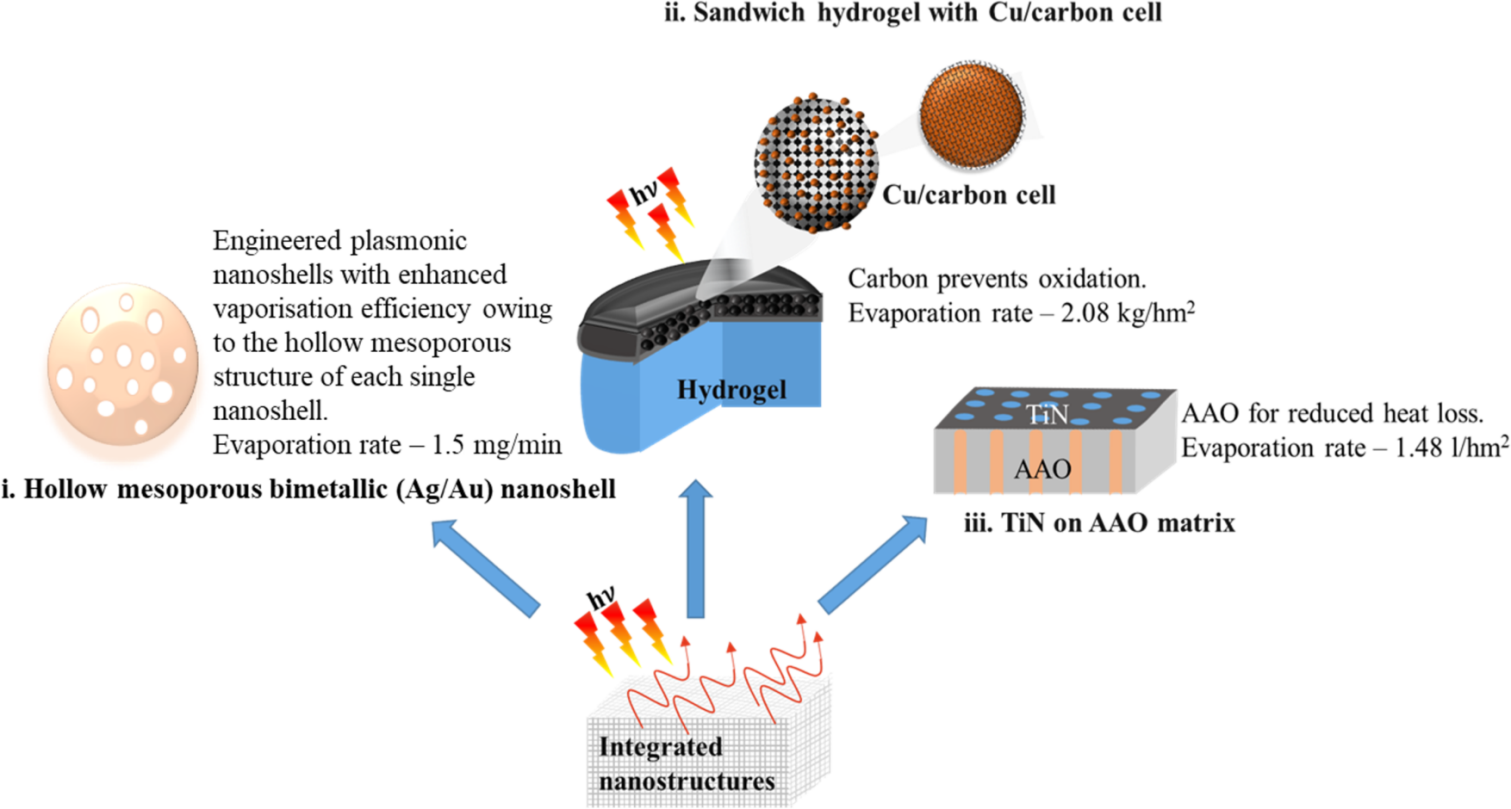
Integrated nanostructures for PT conversion. (i) Hollow mesoporous bimetallic (Ag/Au) nanoshells for solar vapour generation. Figure 23 panel i was redrawn from [169]. (ii) Sandwich hydrogel with Cu/Carbon cell. Figure 23 panel ii was redrawn from [206]. (iii) TiN on AAO matrix. Figure 23 panel iii was redrawn from [207].

Pristine Ag nanoparticles had a steam generation efficiency of 60–80% under an irradiation power of 810–930 W/m^2^ [203]. There was a considerable enhancement after combination with TiO_2_ through the LSPR effect. The efficiency also increased due to an increase in absorbance when the volume fraction of Ag was increased due to an increase in internal scattering within the system [204]. Hence, the plasmon resonance properties of doped metal oxides as well as metal–metal oxide composites may yield new performance benchmarks in PT research [205]. Some of the metal incorporated metal oxide nanoparticles are shown in Figure 23.

#### 4.4 Transition-group plasmonic nanomaterials

**Figure 24:**
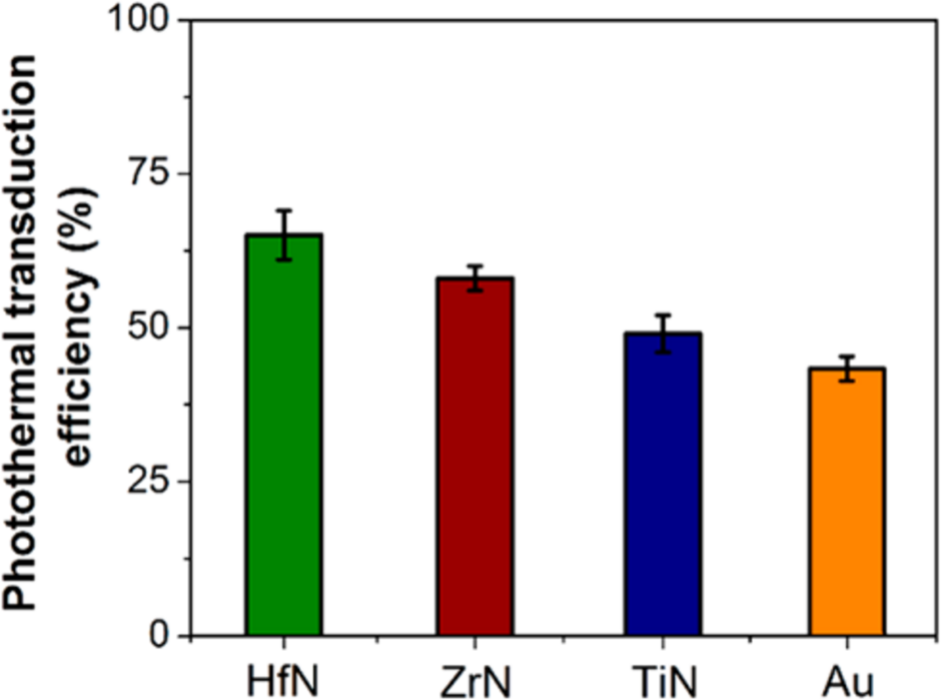
PT conversion efficiencies of different transition metal nitrides (HfN, ZrN, and TiN) and the corresponding efficiency for Au nanoclusters, all at a laser power intensity of 1.0 W/cm^2^. Figure 24 was reprinted with permission from [209], Copyright 2020 American Chemical Society. This content is not subject to CC BY 4.0.

Transition metal nitrides (TMNs), such as ZrN, HfN, VN, NbN, TaN, CrN, MoN and WN, exhibit plasmonic absorption in the UV–NIR region and have been proposed as alternative materials to the conventional metals [208–209]. Ab initio and molecular modelling calculations predict that group-IV and group-VI TMNs have lower hot carrier lifetimes in comparison to group-V TMNs, indicating a stronger electron–phonon coupling and hence a better potential for PT conversion. Research on PT performance of TiN for example, presented it as an efficient solar absorber and the most efficient one among TiN, ZrN, TaN, HfN, and WN due to the higher absorption cross section across the entire solar spectrum. Chemical stability, high melting point, and visible-to-NIR plasmonic absorption (500 to 1100 nm) of titanium nitride nanoparticles make them attractive, and more so when considering that their scattering efficiencies are comparable to those of Au nanoparticles [210]. Although detailed investigations regarding conversion efficiency, damping properties, and morphology optimisation were not carried out, a threefold temperature increase was observed for aggregated composite TiN particles compared to the control sample without any nanoparticles [211]. An illustration of the PT conversion efficiency comparing the nitrides to Au under the same laser fluence validates their superior properties in this regard, as shown in Figure 24.

Interestingly, the absorption wavelength range of TiN is much higher than that of Au, making TiN highly attractive for PT heating applications. This is specifically applicable for PT heating for biological applications, wherein the spectral match between the dipolar resonance of TiN (around 700 nm) and the biological transparency window [212] (650–950 nm) renders it attractive (see Table 5). Detailed investigations of the PT heating of TiN for applications other than for biology are limited. A major limitation of TiN is that it is conventionally synthesized through physical vapour deposition processes such as pulsed laser deposition, reactive sputtering, or molecular beam epitaxy, translating to high equipment and handling cost. This also dictates that they are processed as deposited films, requiring substrates with epitaxial capabilities. Although such substrates are available (Al_2_O_3_ and MgO), there is still a restriction on the final configuration of the PT heating system as it must be compatible with films. The availability of solution-processed and even doped TiN and other transition group plasmonic nitrides is continually being worked on [212–214]. Hence, one can expect new synthesis procedures for highly temperature stable and efficient PT nanomaterials. Combinations of old and new plasmonic materials, each with their own merits in terms of absorption properties, tuning, chemical and thermal stabilities, and broadband absorption, are also being explored, as revealed by studies employing both AuNPs and TiN NPs. The synergistic effect of the resulting composite [197] yielded a better PT heating than either of the pure materials, primarily due to the enhancement of the absorption range of solar radiation. It is evident from the few examples presented here that considerable research effort on plasmonic nanoparticles for PT applications is made, with an unrestricted search on material classes. A detailed compilation of all morphologies and chemistries explored for PT heating [142] is presented in Table 6.

**Table 5:** PT conversion properties for plasmonic metal nitride nanostructures of different morphologies with radius (*R*) and thickness (*T*) using different laser power intensities.

No.	Material	Morphology	Dimensions	Rise in temperature (K)	Irradiation/power	References

1	TiN	nanodiscs	*R* = 180 nm, *T* = 30 nm	5	laser/1 µW	[141]
2	TiN	spheres	*R* = 50–300 nm	86.6	—	[211]
3	TiN	nanodiscs	*T* = 180 nm	29	laser/650 mW	[141]
4	TiN	thinfilm	*T* = 40 nm	1.75	Nd:YAG laser/0.162 mW	[215]
5	TiN	membrane	*T* = 10 mm	54	laser/1 sun illumination	[216]

**Table 6:** PT conversion properties of various classes of nanostructures. *A* denotes the aspect ratio, *R* is the particle radius, and *T* is the thickness.

Material class	Morphology	Dimension	Rise in temperature (K)	Power intensity

metals	spheres	*R* = 20–150 nm	14–573	1–375 mW
	rods	*A* = 4–7 nm	45–55	1 mW
	sheets	*T* = 2–80 nm	4–50	0.1–1 W
	other morphologies	cubes: *a* = 75 nm; sheets: *L* = 41–50 nm	20–227	1–3.8 kW
bimetals	plates	*T* = 5.4–15 nm	50	0.3–1.4 W
	other morphologies	cubes: *a* = 22.8 nm; rods: *A* = 3.5 nm	36–230	2.4–8 W
metal sulfides	spheres	*R* = 5–20 nm	12–19	1.4–40 W
	other morphologies	nanodots: *R* < 5 nm	19	1.41 W
metal oxides/metal–metal oxide composites	spheres	*R* = 300 nm	70	7 kW
	core–shell	core: *R* = 19–62 nm; shell: *R* = 40–77 nm	2–80	1–5 kW
	other morphologies	nanorods: *A* = 3–3.3 nm; thin film: *T* = 164–150 nm	6–230	1–10 kW
metal nitrides	nanodiscs	*T* = 30–180 nm	5–29	1 µW–650 mW
	other morphologies	spheres: *R* = 50–300 nm; thin film: *T* = 10–40 nm	54–86	1 mW

We have graded the performance in PT applications of the materials elaborated in Table 6. Evidently, morphologies other than spheres can offer significant enhancements to PT performance. This can be due to enhancements to the damping of the resonance (due to scattering with the surface in case of 2D nanostructures/confined nanostructures), enhanced surface area for irradiation, and reduced tendencies for agglomeration. Combined with the effects of plasmonic field coupling on the resonance properties, opportunities abound with respect to the possibilities of employing a combinatorial approach of such morphologies for achieving improved PT performance.

### 5 Stability of PPT materials

The definition of nanoparticle stability depends on the desired property that is to be preserved. For catalytic applications, stability pertains to the preservation of the surface area/active crystal facets, whereas for applications such as light harvesting, the active area shares its importance with the necessity of preservation of the optical absorbance properties. Since for PT applications, the latter is more important, stability of nanoparticles for PT applications in terms of morphology and composition is most important, as the optical properties are determined by them, and should be maintained under environments of temperature, pressure, and chemically active atmospheres present in various PT applications [217]. Chemically active atmospheres can, for example, result in a quenching of the plasmonic resonance as a result of strong interactions between adsorbed species and the plasmonic nanoparticle. Anisotropic morphologies such as nanorods [218] or stars [219] can be very susceptible to high temperature, resulting in rounding with a loss of anisotropy and loss of the plasmonic absorbance. Doped plasmonic nanoparticles are particularly vulnerable to temperature and reactive atmosphere effects, due to diffusion, segregation, or reaction of the dopant ions resulting in conversion to a nonplasmonic composition.

More often than not, absorbance tuning of plasmonic nanoparticles is done by effecting a change in the refractive index of the medium, that is, by surrounding the NPs with different materials to form structures such as core–shell [220], hierarchical [90], or decorated structures [221]. Thus, the stability of the encapsulating material also becomes crucial, as a loss of stability can result in the loss of the tailored absorbance. As an example, highly defect-rich nanoparticles such as twinned/icosahedral structures [222] are very susceptible to chemical attacks (e.g., by O_2_ or salts) and have been reported to be rapidly dissolved, losing all effectiveness for PT heating. Different stability aspects of nanoparticles that affect their optical absorbance will be presented subsequently.

#### 5.1 Aggregation stability of nanoparticles in liquid-phase PT heating

**Figure 25:**
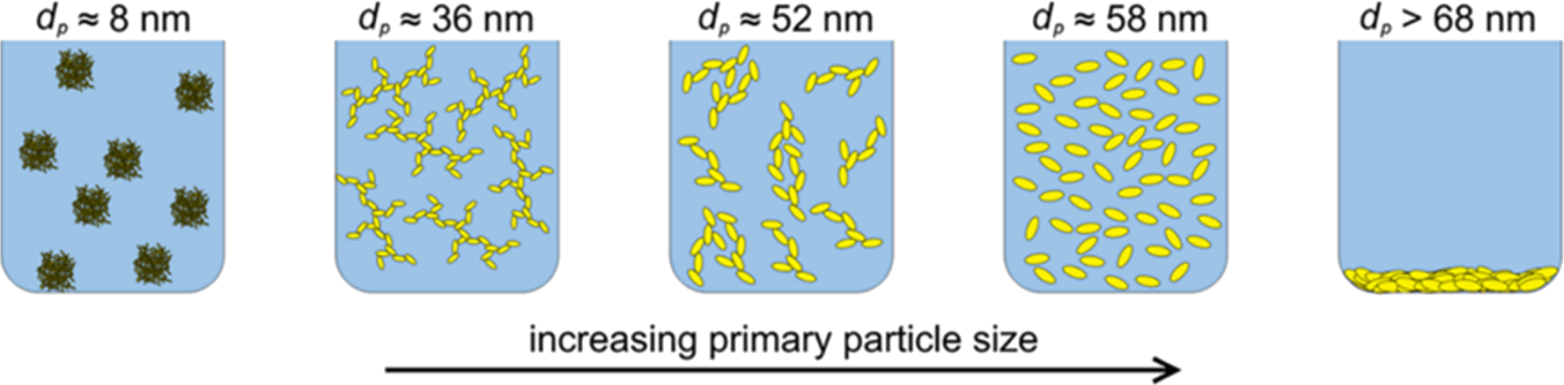
Colloidal stability of nanoparticles for different particle sizes from 8 to 68 nm of organic pigment samples. Figure 25 was reprinted with permission from [224], Copyright 2017 American Chemical Society. This content is not subject to CC BY 4.0.

A common approach to exploit the PT heating effect of plasmonic nanoparticles is to disperse them in PCM hosts, which absorb and store heat during a phase change, such as from solid to liquid or solid to solid. The most common mechanism through which nanoparticles added to liquid PCMs lose their properties is by aggregation (Figure 25) during storage or during the phase change process. Aggregation stability depends on the collision frequency between the particles and the interaction pair potential between them (a summation of the van der Waal’s potential, electrostatic interaction potential, osmotic potential, and the elastic potential), and on whether this potential exceeds a kinetic energy barrier. To obtain this maximum energy barrier, the collision frequency, dictated by the number density of the nanoparticles, and their root mean square velocity ⟨*v*_rms_⟩ is calculated first by [223]:


[44] $$\langle V\rangle ={\left(\frac{8k_{\text{B}}T}{\pi \mu }\right)}^{0.5},$$

where *k*_B_ is the Boltzmann constant, *T* is the temperature, and μ is the reduced mass of two objects.

From the resulting number of collisions per unit time and knowing the desired time over which stability against aggregation is required, the number of collisions during this time can be calculated. Assuming a lower probability of collisions than this over the desired time period will allow for the determination of the threshold barrier for aggregation. Nanoparticle aggregation depends on whether the collisions between the nanoparticles are elastic or inelastic. Elastic collisions result in the colliding nanoparticles remaining separated, while inelastic collisions result in nanoparticle aggregation. Hence, inelastic collisions result when the interaction pair potential exceeds the barrier (*V*_max_) and nanoparticle aggregates are formed [225]. As an example, *V*_max_ for nanoparticles that need to be non-aggregated for a period of one week has been calculated as [223,226]


[45] $$V_{\max}=16k_{\text{B}}T.$$

Thus, the stability of nanoparticles against aggregation can be determined by such calculations, and their handling can be modified accordingly by changing conditions such as pH or by introducing capping ligands/stabilizers [227]. It is also interesting to note that in the case of highly anisotropic nanostructures, such as nanostars, the smallest dimension of the nanostructure in solution determines its agglomeration stability [223].

#### 5.2 Chemical stability of nanoparticles in PT heating

**Figure 26:**
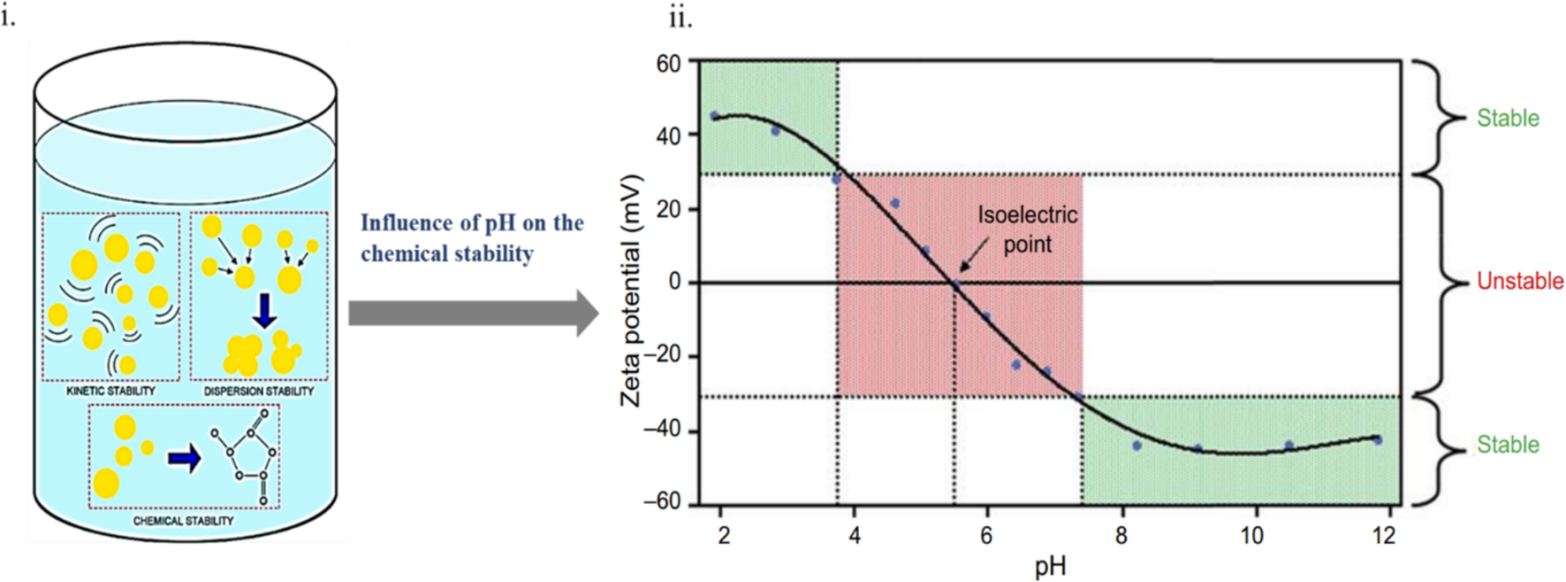
Illustration of the chemical interaction stability of nanoparticles. (i) Chemical interactions between fluid/particle dictate chemical stability. Figure 26 panel i was reprinted from [228], Nanofluids for Heat and Mass Transfer, 2021, by B. Bhanvase; D. Barai, “3 - Stability of nanofluids”, pages 69-97, Copyright (2021), with permission from Elsevier. This content is not subject to CC BY 4.0. (ii) Impact of pH value on stability of nanoparticles, as can be visualized through the zeta potential. Figure 26 panel ii was reprinted from [229], Advances in Chemical Mechanical Planarization (CMP) (Second Edition), 2021, Babu, S., Ed.; by K. Pate; P. Safier, “13 - Chemical metrology methods for CMP quality”, pages 355–383, Copyright (2022), with permission from Elsevier. This content is not subject to CC BY 4.0.

It is obvious that the high surface area of nanoparticles leads to a weak chemical stability, which is conventionally viewed as the inherent resistance to irreversible reactions. This resistance is crucial for preventing loss of optical properties. Chemical stability, like agglomeration stability, is influenced by kinetics as well as thermodynamics as shown in Figure 26. Kinetic stability is determined by the ionization energy. As an example, Ag is more easily oxidized than Au due to its lower ionization energy.

Thermodynamic stability of the phase of a nanoparticle is determined by the free energy of formation of its state. Since an exhaustive compilation of the preferred states of nanoparticles is not the focus of this work, only one example of the thermodynamic stability of Ag is considered. The oxidation of Ag has a free energy of formation of −11.25 kJ/mol [225]. With a change in nanoparticle size (e.g., for tuning the optical absorbance), the free energy of formation is influenced even at 25 °C [225]:


[46] $$\Delta G_{298}^{{}^{\circ}}\left(r\right)=-11.25\text{ kJ }{\text{mol}}^{-1}-\frac{57.5\;{\text{kJ mol}}^{-1}\text{ nm}}{r},$$

where *r* is the radius of the nanoparticle in nanometres. Yet another plasmonic metal that is highly promising for ultraviolet PT conversion is aluminium. However, aluminium is easily oxidized with consequent loss of the plasmonic absorbance. This problem is even stronger when aluminium is directly exposed to water because of the significantly enhanced oxidation rate (forming hydrates). Interestingly, the tuning of the nanoparticle dimensions can influence this stability against oxidation. As an example, lithographic aluminium nanodiscs of 100 nm diameter showed a remarkably sustained plasmonic resonance even after a year, compared to larger and smaller discs where the resonance disappeared within days. The reduced grain boundary coarsening [230] of these specifically sized discs due to self-annealing was the reason behind the reduced oxidation rates.

It stands then that nanoparticle chemical stability changes with size/morphology/encapsulation. Nanoparticles can be added with morphologically controllable exterior layers of functional ligands to prevent access of potentially harmful species, such as functionalizing with thiosulfate ions [231], use of conventional surfactants and ligands such as PVP [232], CTAB [233] and citrate [234], and biomolecular ligands such as DNA [235] (due to the strong negative charge on its phosphate backbone) and proteins [236]. Shells composed of organic [237], inorganic [238], or metallic [239] materials are also an option. Another approach to enhance chemical stability is to add dopants such that charge transfer and stabilization through the dopant are achieved. An example is the addition of Ag or Au to stabilize Cu in its pristine [240] form, morphological transformations notwithstanding. Thus, approaches of encapsulation and/or doping can stabilize plasmonic nanoparticles.

The major shortcomings of these approaches that hinder their application in PT heating are the changes to the optical absorbance of the primary nanoparticle as a direct result of the doping or encapsulation processes. These changes include quenching, broadening or narrowing of the absorption, shifts to different wavelengths, as well as changes to other PT-relevant properties such as thermal conductivity, especially in the case of the encapsulation. Changes to thermal conductivity due to encapsulation can be a bigger concern than doping, as loss of thermal conductivity due to phonon scattering at the interface of the encapsulating layer will be present for these materials, but not necessarily after doping. A careful selection of the appropriate methodology for enhancing the chemical stability is therefore essential.

#### 5.3 Thermal stability of nanoparticles for PT applications

**Figure 27:**
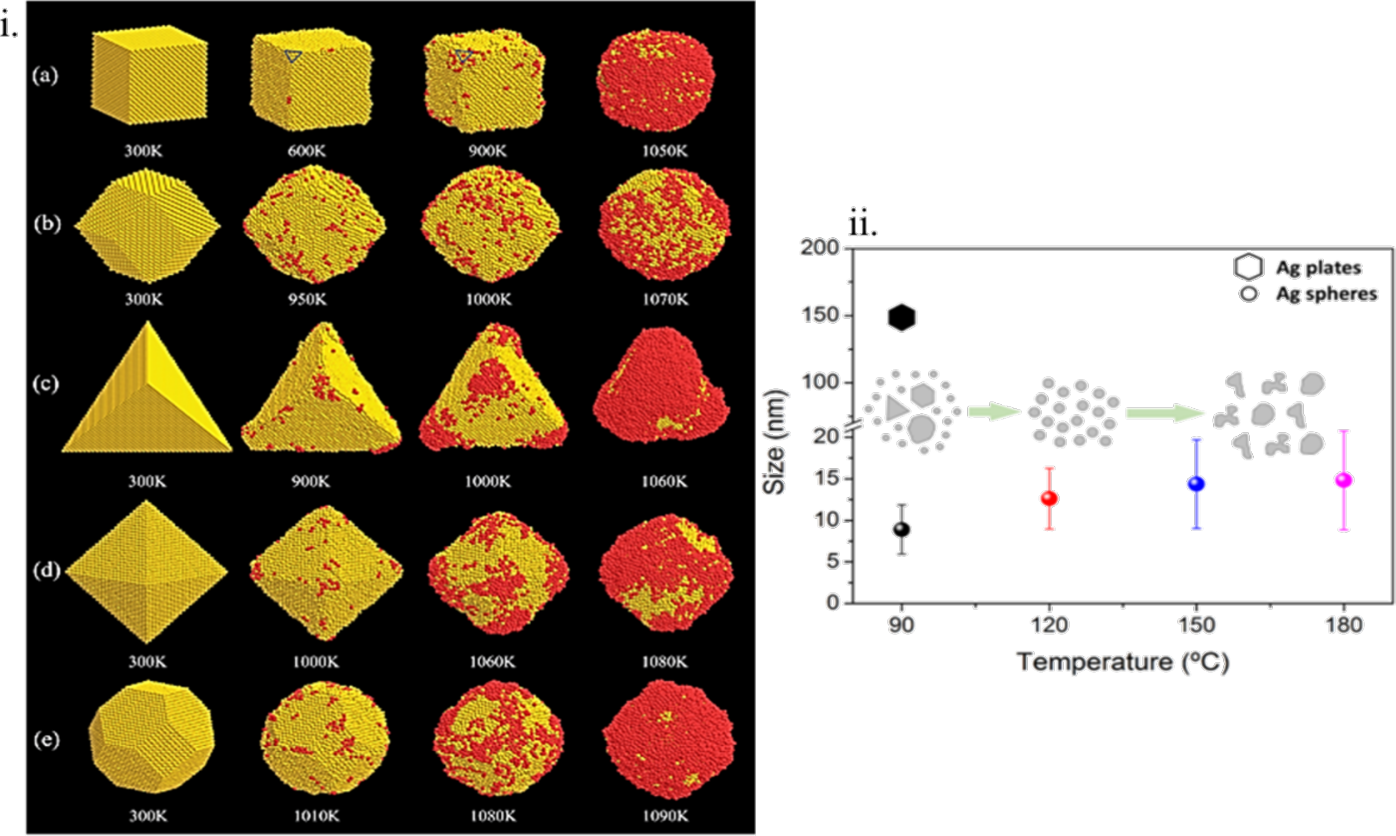
Thermal stability of Ag and Au nanoparticles of different shapes at different temperatures. (i) (a) Au nanocube, (b) rhombic dodecahedron, (c) tetrahedron, (d) octahedron, and (e) truncated octahedron. Figure 27 panel i was used with permission of The Royal Society of Chemistry, from [222] (“Single-crystalline and multiple-twinned gold nanoparticles: an atomistic perspective on structural and thermal stabilities”, by R. Huang et al., RSC Adv., vol. 4, issue 15, © 2014); permission conveyed through Copyright Clearance Center, Inc. This content is not subject to CC BY 4.0. (ii) Ag nanospheres and Ag nanoplates of different sizes that can be realized as a function of temperature. Figure 27 panel ii was reprinted with permission from [244], Copyright 2020 American Chemical Society. This content is not subject to CC BY 4.0.

**Figure 28:**
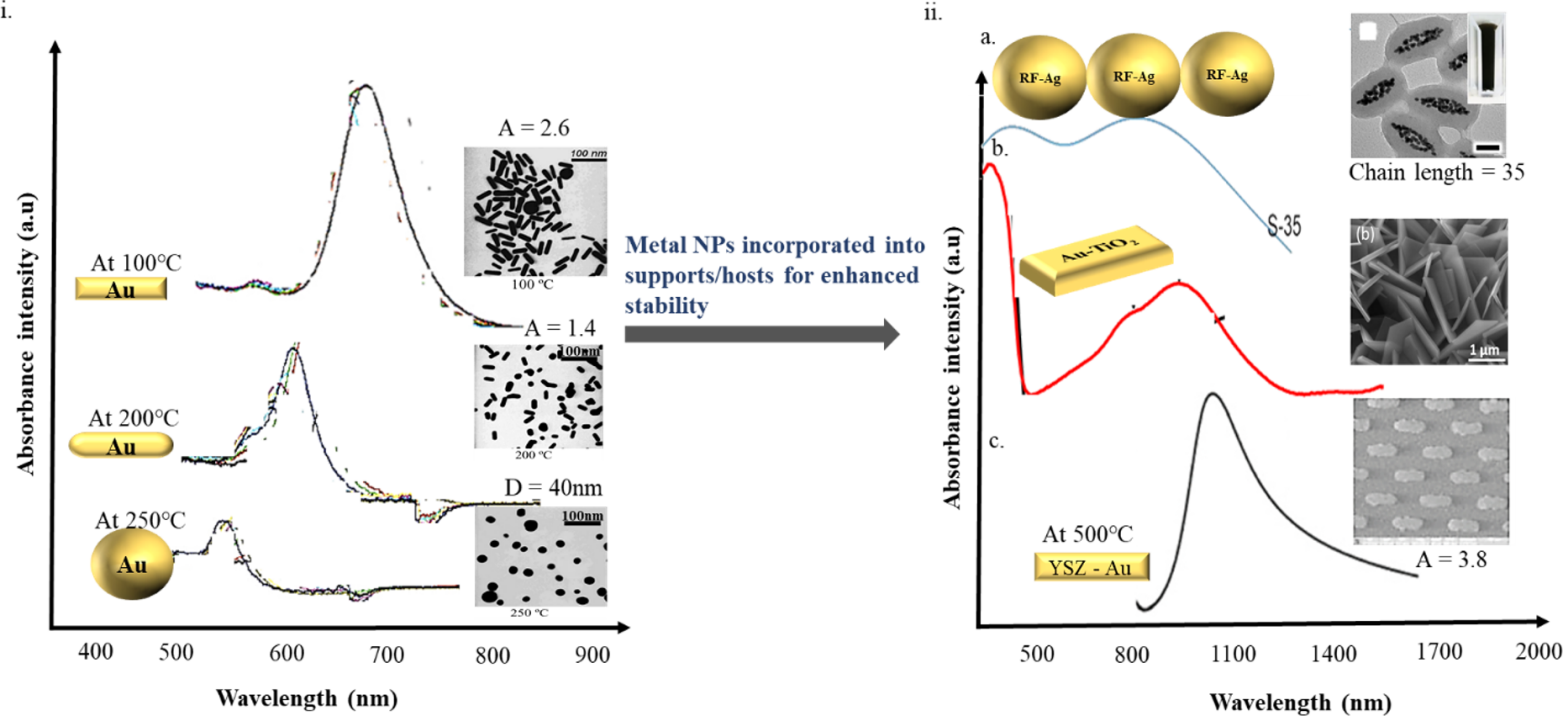
Thermal stability of Au nanorods at increasing temperatures. (i) Aspect ratio and plasmonic absorbance spectra of Au nanorods shift with increasing temperature. Figure 28 panel i was adapted with permission of The Royal Society of Chemistry, from [245] (“On the temperature stability of gold nanorods: comparison between thermal and ultrafast laser-induced heating”, by H. Petrova et al., Phys. Chem. Chem. Phys., vol. 8, issue 7, © 2006); permission conveyed through Copyright Clearance Center, Inc. This content is not subject to CC BY 4.0. (ii) (a–c) To prevent this, AuNPs can be incorporated into support materials such as yttrium-stabilized zirconia, titania, or resorcinol formaldehyde (RF). Figure 28 panel ii a was adapted with permission from [114], Copyright 2019 American Chemical Society. This content is not subject to CC BY 4.0. Figure 28 panel ii b was adapted with permission from [246], Copyright 2017 American Chemical Society. This content is not subject to CC BY 4.0. Figure 28 panel ii c was adapted with permission of SPIE from [218] (“Investigation of the optical and sensing characteristics of nanoparticle arrays for high temperature applications”, by G. Dharmalingam and M. A. Carpenter, Sensors for Extreme Harsh Environments II, vol. 9491, 13 May 2015, article no. 949108, © 2015 SPIE); This content is not subject to CC BY 4.0.

As discussed in the previous sections, the nanoparticle morphology and the environment control the optical absorbance. Any changes to these factors hence can be detrimental to the suitability of these particles for the concerned purpose. The change in surface energy is a driving force for shape changes in nanoparticles. Despite preservation of the desired optical properties due to controlled synthesis, post-synthesis shape changes can happen through agglomeration (during storage or prolonged use), chemical reaction, and (often more frequently) thermal factors such as increasing temperature. Indeed, the latter mechanism has been developed as a nanomaterial fabrication technique on its own, based on the PT effect and termed as light-induced rapid annealing (LIRA [142,241]). A well-established mechanism of particle size increase due to temperature is the phenomenon of Ostwald ripening, wherein atoms on the surfaces of smaller particles dissolve and deposit on the more stable surfaces of larger particles. This mechanism has not been conclusively proven for nanoparticles dispersed in a liquid phase, although particle growth due to agglomeration presents a significant challenge. For nanoparticles dispersed in a solid matrix (such as the case of solid PCMs), this process can be inevitable without the presence of stabilizing agents/encapsulating layers. Examples of this phenomenon include Pd nanoparticles ripening at ambient temperature [242] as well as at high temperatures of 750 K [243]. Heat trapping layers can be added to achieve plasmonically enhanced PT efficiency and to increase the thermal stability of the plasmonic materials, while also reducing oxidation. For example, Al_2_O_3_ has been added as a trapping layer on Ag to increase the stability to over three months, while Ag alone underwent oxidation within 20 days [198]. The thermal stability of Au/Ag nanoparticles with different morphologies is shown in Figure 27 and Figure 28.

Temperature-driven interdiffusion influences significantly the thermal stability of a nanomaterial, especially when present as alloys or as doped materials. The Kirkendall effect [247] dictates that the interface between two materials having different diffusion rates moves as a function of the temperature as well as the nature of the environment. The result is the formation of new chemistries as well as morphologies. The Kirkendall effect can be envisioned to be a positive or a negative mechanism, wherein the positive aspect is the possibility to achieve novel morphologies such as nanoboxes, fullerene-like nanoparticles [248], and porous structures [249] through facile processes. The negative aspect then is the loss of PT properties due to changes in chemistry and/or morphology even at room temperature. Hence, the knowledge of the diffusion rates of materials is important when employing multiple compositions/phases during the design of PT plasmonic nanomaterials. An evident, but often infeasible, solution to avoid interdiffusion is the storage and handling of the prepared nanomaterials at low temperatures.

Thermal stability of a nanomaterial in PT heating depends on the melting point as well. The diffusion of atoms becomes unrestricted on reaching this temperature at any location within the nanoparticle. Melting is often explained as liquefaction when the amplitude of atomic vibrations exceeds a fraction of the interatomic spacing. The influence of temperature on this amplitude and hence on melting was established as early as 1910 by the relation [250]


[47] $$m{\left(2\pi \nu_{\text{E}}\right)}^{2}{\left(\delta x\right)}^{2}=k_{\text{B}}T,$$

where *m* is the atomic mass, ν_E_ is the Einstein frequency, δ*x* is the root-mean-square thermal average amplitude of vibration, *k*_B_ is the Boltzmann constant, and *T* is the absolute temperature. For nanomaterials, the melting temperature is closely associated with the particle size and given by the relation [251]


[48] $$T_{\text{m}}=T_{\text{mbulk}}\left(1-\frac{C}{D}\right),$$

where *T*_m_ and *T*_mbulk_ are, respectively, the melting temperature of the nanoparticles and bulk material, *D* is the diameter of the nanoparticles, and *C* is a parameter that depends on the model applied for the melting process, that is, the homogeneous melting hypothesis (HMH), the liquid skin melting (LSM) model, or the liquid nucleation and growth (LNG) model [252]. This Gibbs–Thompson relation has been used successfully in multiple studies for estimating the depression in melting point with the size of nanomaterials. It is to be noted that although this model is considered sufficiently accurate by multiple studies, variations of modelling the melting temperature exist, depending on how the melting point is arrived at, such as by modelling the Lennard–Jones potential [251], or whether the microscopic variations in thermal properties and aspects such as shape effects [91,253] are included in the calculations. An example of alternative studies that have successfully modelled nanoparticle melting is the study of the effect of laser irradiation on Au nanorods, wherein the model could predict the temperatures that cause reshaping of the nanorods (due to plasmonic heating) [143]. Temperatures above 1000 K at the irradiation location reshaped the nanorods, while the lower temperatures at the boundaries of the beam spot (795 K) did not affect them [14]. In a representative study, the significantly higher melting point of palladium nanoparticles led to a better performance and maintenance of morphology (and hence the LSPR property) compared to Au and Ag nanoparticles under radiation with NIR light, as detailed in prior sections [119]. The water evaporation rate of the confined linked Ag–Au nanoparticles remained stable after ten cycles while the morphology also remained stable, demonstrating the potential for encapsulation in enhancing nanoparticle stability for PT applications [114].

Platinum nanoparticles normally absorb in the UV–visible range with extensions into the NIR region [115]. Observations such as the melting of the adjacent areas of a NIR-irradiated Pt nanoparticle at temperatures greater than 700 °C and the stability of the nanoparticle itself leads to the postulation that they can be viable candidates for PT heating applications. While the process of laser irradiance for studying plasmonic heating is the rational approach to obtain precise information on the absorbance properties, it must be kept in mind that an extremely localized heating of the nanoparticles or the nanoparticle dispersion occurs in these studies. This observation, although accurate for applications such as photodynamic therapy, does not reflect accurately the processes such as water vaporisation through solar power irradiation, wherein the irradiation area is considerable higher than a micro-/millimetre-sized laser beam. This difference in the heating process is important as it can lead to very different outcomes. Nanoparticles to be used for solar heating studied by laser irradiation will not necessarily melt and undergo morphology changes when eventually deployed in practice. Hence, it is crucial that nanomaterials to be employed for PT conversion applications are characterized by accurately reproducing the field deployment guidelines.

The discussion of the stability of nanomaterials for PT energy conversion thus takes into account the predominant reasons affecting the same, namely temperature, chemical reactions, and aggregation. Regardless of the phase of the PT conversion system (liquid/solid/gas), these three phenomena govern the suitability as well as the life cycle of the nanomaterial. Proper guidelines that can be derived from the application need to be considered for the selection and design of PPT nanostructures for optimal performance.

## Conclusion and Outlook

Plasmonic nanoparticles are definitely a topic in PT research, be it in medicine for therapy or regarding energy for storage, heating, or chemical syntheses. We have presented a review on established as well as emerging plasmonic materials that are of importance for a multitude of applications. Among the principal requirements for a material for PT research is the conversion efficiency. It is clear that among the materials presented, the conversion efficiency of pristine materials is markedly lower than that of composited materials, for reasons such as non-ideal dielectric properties (for heating), performance degradation over time, limited possibilities in terms of the absorption wavelength range, and stability against aggregation, reaction, or temperature. Thus, materials composed of multiple elements and/or morphologies are needed to advance the practicality of PPT materials. Novel morphologies such as linked nanoparticles, collapsed nanowires, and concentric rings composed of multiple materials or even the simple polydispersed nanoparticles can lead to vastly better PT properties in terms of enhancing the absorption range and improving the conversion efficiency. Interesting properties such as optical duality observed in, for example, Te can be exploited to achieve broadband absorption of light through the manifestation of both plasmonic and Mie resonances. We have reported on a narrow hybrid resonance mode, which arises due to coupling of the plasmonic and a second diffuse mode on an underlying metal oxide layer. Thus, hybrid (or) multiple-resonance materials can be promising for PT research. A minor shortcoming of such combinations is the inadequacy of simulation methods to comprehensively predict the optical properties. Often chemical reactions that might occur when bringing such materials together are not known and cannot be included in the simulations. Nevertheless, simulations can help in understanding discrepancies between expected and observed material behaviour. It is the authors’ opinion that a major hindrance to broadening the spectrum through combining materials for achieving optimal plasmonics is the stability or lack thereof when these materials are brought together. Deep understandings of the aspects which lead to instabilities, that is, aggregation, irreversible reaction, and thermal degradation can help in this regard. This is also valid for the unexpected interplay between grain size and the enhancement in PT property preservation. With continual pursuits on improving the presented aspects, plasmonic materials can be expected to drive PT research towards a better future.
